# Roadbumps at the Crossroads of Integrating Behavioral and *In Vitro* Approaches for Neurotoxicity Assessment

**DOI:** 10.3389/ftox.2022.812863

**Published:** 2022-02-25

**Authors:** G. Jean Harry, Sandra McBride, Shannah K. Witchey, Sakina Mhaouty-Kodja, Alain Trembleau, Matthew Bridge, Anna Bencsik

**Affiliations:** ^1^ Neurotoxicology Group, Molecular Toxicology Branch, Division National Toxicology Program, National Institute of Environmental Health Sciences, Durham, NC, United States; ^2^ Social & Scientific Systems, Inc., a DLH Holdings Company, Durham, NC, United States; ^3^ Division National Toxicology Program, National Institute of Environmental Health Sciences, Durham, NC, United States; ^4^ Sorbonne Université, CNRS, INSERM, Neuroscience Paris Seine – Institut de Biologie Paris Seine, Paris, France; ^5^ Sorbonne Université, CNRS UMR8246, Inserm U1130, Institut de Biologie Paris Seine (IBPS), Neuroscience Paris Seine (NPS), Paris, France; ^6^ Anses Laboratoire de Lyon, French Agency for Food, Environmental and Occupational Health & Safety (ANSES), Université de Lyon 1, Lyon, France

**Keywords:** behavioral toxicity, developmental neurotoxicity, new approach methodologies, behavioral phenotype, *in vitro* model neurotoxicity, mechanistic neurotoxicity, neurobehavioral screening, neurotoxicity screening

## Abstract

With the appreciation that behavior represents the integration and complexity of the nervous system, neurobehavioral phenotyping and assessment has seen a renaissance over the last couple of decades, resulting in a robust database on rodent performance within various testing paradigms, possible associations with human disorders, and therapeutic interventions. The interchange of data across behavior and other test modalities and multiple model systems has advanced our understanding of fundamental biology and mechanisms associated with normal functions and alterations in the nervous system. While there is a demonstrated value and power of neurobehavioral assessments for examining alterations due to genetic manipulations, maternal factors, early development environment, the applied use of behavior to assess environmental neurotoxicity continues to come under question as to whether behavior represents a sensitive endpoint for assessment. Why is rodent behavior a sensitive tool to the neuroscientist and yet, not when used in pre-clinical or chemical neurotoxicity studies? Applying new paradigms and evidence on the biological basis of behavior to neurobehavioral testing requires expertise and refinement of how such experiments are conducted to minimize variability and maximize information. This review presents relevant issues of methods used to conduct such test, sources of variability, experimental design, data analysis, interpretation, and reporting. It presents beneficial and critical limitations as they translate to the *in vivo* environment and considers the need to integrate across disciplines for the best value. It proposes that a refinement of behavioral assessments and understanding of subtle pronounced differences will facilitate the integration of data obtained across multiple approaches and to address issues of translation.

## 1 Introduction

The nervous system is comprised of a dynamic interactive circuitry involving communication between neurons, glia, neurovascular and neurolymphatic systems for which temporal and spatial regulation are critical factors. It is responsible for transmitting information about the environment and communicating and integrating that information to respond to and operate on that internal or external environment. In this response, behavior is context-dependent and, while it can be altered by variations in cellular or physiological events, it is dynamically shaped by experiences and reinforcements. With the appreciation that behavior represents the integration and complexity of the nervous system, the field of neurobehavioral phenotyping and assessment has seen a renaissance in neuroscience research over the last decade. This has produced a robust database on rodent performance within various testing paradigms, possible associations with human disorders, and therapeutic interventions. Thus, multiple tracts for behavioral assessment have evolved including, phenotyping of genetic manipulation, readouts of specific disorders, neurotoxicity evaluations of pharmaceutical or chemical agents, and understanding underlying mechanisms associated with sensory, motor, cognitive/learning performance, and memory.

That drugs or chemicals in the environment might adversely affect the nervous system has been a general concern for years with emphasis on the vulnerability of the developing nervous system. Epidemiological literature on childhood effects of neurotoxicants is often difficult to assess due to the complex nature of brain functions, the multiple factors that influence brain development, and exposure to multiple environmental factors. Similarly, with the complex lifetime exposome profile, linking a causative effect of exposure and neurodegenerative diseases is limited. Data from experimental animal studies provide a basis for confirming that exposure to chemicals and physical factors can have adverse consequences on the nervous system. Such data is available to implicate effects on the developing brain that may result in long-term consequences or latent effects that manifest later in life. More recently, data is available demonstrating alterations in healthy aging and the susceptibility of the aged nervous system to insult.

**FIGURE 1:**
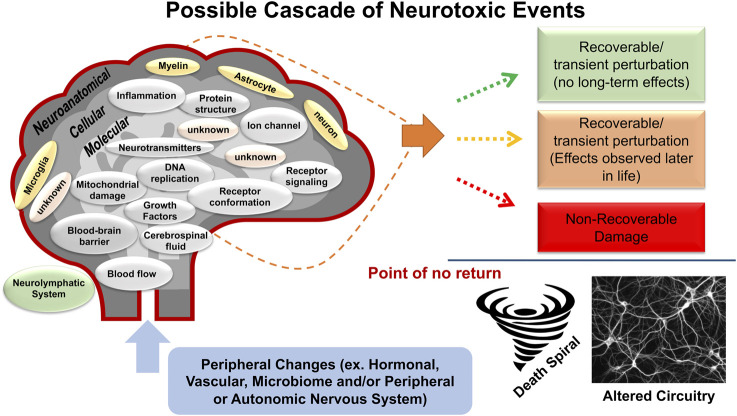
Schematic representation of possible cascade of neurotoxic effects. The schematic represents a cascade of neurotoxic events that can occur following exposure to a chemical or physical agent. These events form the foundation of the definition of *in vivo* neurotoxicity “an adverse change in the structure or function of the central nervous system and/or peripheral nervous system”. They address many of the points surrounding the concept and definition of “adverse” in that they reflect the multitude of cellular and molecular changes that can occur to alter the function and susceptibility of the nervous system. Effects can occur by direct chemical exposure and more indirectly by alterations in the peripheral and autonomic nervous systems, in the periphery (i.e., hormonal, vascular, microbiome, etc.), in the specialized protective (e.g., blood-brain-barrier) and drainage (cerebral spinal fluid, neurolyphatic) systems. Additionally, the read-out of these effects can manifest differently. Three scenarios are proposed. 1) the insult is relatively short-term and there is recovery from a transient perturbation with no long-term effects. 2) the insult is recoverable but there are latent effects that manifest later in life. The transient nature can be due to an active process to return the system to homeostasis through adaptation mechanisms. While there are no apparent long-term effects, the “adapted” system may not necessarily reflect a return to normal. This is reflected in the second outcome the exposure-related effects may manifest later in life. 3) There is non-recoverable damage that can range in severity from an alteration in the neural circuitry and signaling capability to cell death. While evidence of neuropathology is clearly indicative of a neurotoxic outcome, the absence of neuropathology does not indicate an absence of neurotoxicity.

A fundamental tenet of pharmacology is that all drugs will have multiple effects, this is even more true for environmental factors and chemicals and will likely be demonstrated with genetic modifications. In assessing *in vivo* neurotoxicity, a framework has been set with the definition for neurotoxicity of an adverse change in the structure or function of the central and/or peripheral nervous system following a biological manipulation (US EPA, 1994a; US EPA, 1994b; US EPA, 1998a; US EPA, 1998b; IPCS, 2001). While neuropathology could be an outcome, neurotoxicity can often be the result of numerous processes in the absence of overt neuropathology (Norton, 1978; US EPA, 1998a; US EPA, 1998c; IPCS, 2001). The operational definition of adverse includes any alteration from baseline functioning that diminishes an organism’s ability to survive, reproduce, or adapt to its environment. This may be a change in morphology, physiology, growth, development, or aging that results in an impairment of functional capacity, an impairment of the capacity to compensate for additional stress, or an increase in susceptibility to other environmental influences. The schematic in Figure 1 represents the diverse targets of neurotoxicity and the possible outcomes.

The importance of screening for neurological effects of chemical exposure has been evident since the mid-1970s, with further expansion and refinement seen in the 1980s and 1990s, largely based on evaluations of sensitivity, reproducibility, and comparability (Harry et al., 1995; Tilson, 2002). At a workshop in 1989 to evaluate the qualitative and quantitative comparability of several human and animal developmental neurotoxicants (lead, drugs of abuse, alcohol, PCBs, phenytoin, methylmercury, and ionizing radiation), it was concluded that there was a considerable comparability of end-points across species; that animal testing methods measured qualitatively similar effects in humans; and that quantitative differences between animals and humans was based largely on differing toxicokinetic factors (Rees et al., 1990). The comparability of qualitative changes was most evident when made on general categories of behavioral functions (motor, sensory, learning/memory) rather than upon specific individual tests. This is consistent with findings from a multi-laboratory study on strain differences concluding that larger differences were replicated across lab while those of moderate effect sizes showed greater variability (Wahlsten et al., 2003). For quantitative comparability, it was reported that a measure of internal dose was required. Over the years of evaluating *in vivo* test methods for detecting developmental neurotoxicity, sensitivity of the methods has been confirmed by numerous panels of experts [review, (Tilson, 2002)]. Thus, early in the establishment of standardized tests for neurotoxicity there was an appreciation of the limitations of stand-alone individual tests or limited endpoints and that target tissue levels were necessary for quantitative comparisons.

A more recent concern of various regulatory agencies tasked with integrating such data into risk assessment is based on the quality of the data submitted for regulatory review. The various regulatory agencies proport that the tests, as conducted according to the standard testing guidelines, and the data presented, appear to be highly variable and of limited utility for risk assessment (Makris and Vorhees, 2015). While the default position appears to be that behavioral assessments are inherently variable, there are additional contributory issues that have been raised such as the absence of standardized testing protocols and data analyses or consideration of dose selection for evaluation. One primary concern is related to the question of whether the utility of the assays are due to limitations of the tests or rather to the conduct of “standardized test” by investigators with limited fundamental formal training in behavioral assessments and/or learning and memory. To address the issue of test quality and consistency, a number of guidance documents, protocol publications, and data analysis papers have been published over the years (IPCS, 1986; Slikker et al., 2005; Bailey et al., 2006; Moser, 2011; Hånell and Marklund, 2014; NTP, 2015; NAFTA, 2016; Saré et al., 2021; Vorhees and Williams, 2021). Yet, even with these protocols and commercially available equipment, there are questions on the transition of such optimized procedures to protocols amenable to a larger contract testing environment with the capacity to assess a large number of animals.

With the establishment of “guideline studies” for regulatory decisions and the commercial availability of equipment, the emphasis shifted out of the academic laboratory and expertise in the specific type of test, to a broad screening environment. With this shift comes the potential for the generation of large amounts of data but a loss of expertise in the neurobiology of behavior for quality assessments, data analysis, and interpretation of the findings. A third consideration is outside the actual behavioral test itself but rather is based on expectations placed on the tests by the regulatory community with regards to qualitative vs. quantitative assessments (Tilson, 2002; Wahlsten et al., 2003). These multiple factors have contributed to the quality of data and the perception that apical endpoints and their inherent individual variability are not sufficiently sensitive for regulatory purposes. The perceived issues with *in vivo* apical assessments have led to a proposal to transition to a more reductionist approach and/or use of less complex *in vivo* models to screen for neurotoxicity. These include various *in vitro* model systems primarily focused on neuronal cells and non-mammalian model systems such as, zebrafish and *C. elegans*. Many of these model systems are likely covered in accompanying manuscripts in this special issue and have been extensively presented in multiple recent publications (Schmidt et al., 2017; Pistollato et al., 2020; Pistollato et al., 2021; Sachana et al., 2021). However, with this transition comes the need to formulate specific experiments to demonstrate validity of the assays to represent, *in vivo*, the proposed underlying biological process. This brings the field to a crossroad. Identifying the benefits and limitations of each approach, developing a strategy for integrating *in vivo* and *in vitro* studies and findings, inclusion of mechanistic endpoints, and providing validation to support translation to *in vivo*, and prediction to an adverse health outcome are necessary to fully advance the field and to ensure a level of confidence in the data for human health risk assessment (Carlson et al., 2020; Payne-Sturges et al., 2021).

One step in this process is to undertake an honest consideration of various pitfalls and missed opportunities of the rodent behavioral studies and to learn from this to minimize similar “failures” in any future approaches, *in vivo* or *in vitro*. It is thought that a better understanding of the apical endpoints and the efforts needed to refine the assessments will be a step in that direction. It will also be a necessary step in any future effort to translate findings from *in vitro* experiments to adverse effects *in vivo*. The current manuscript reviews the background for inclusion of neurobehavioral assessments in neurotoxicity assessments, various considerations, and historical evaluation of such studies, statistical approaches, and data presentation. It is not meant to be all inclusive of behavioral assessments but rather to present aspects needed for quality assessments. In concordance with additional manuscripts in this issue, consideration is given to how to integrate *in vitro* model systems within the framework of benefits and limitations. How they can identify potential target cells and enhance and embellish our understanding of modes of action for neurotoxicity.

## 2 Validity and Reproducibility of Test Methods

### 2.1 Validity

In any model development, *in vivo* or *in vitro*, a critical evaluation of the model under study is needed to warrant further investment. While model validity is often asserted by the investigator, a discussion on the type of validity and the terms of strengths and weaknesses is required. A longstanding framework posits three types of validations: construct, face, and predictive. Construct validity refers to the degree to which a test measures what it claims to measure. As this applies to neurological disorders, an animal model would demonstrate the etiological processes that cause the disorder. For an *in vitro* model, construct validity can take on two faces, one as to whether it measures the specific *in vitro* endpoint it proports to measure or in the next demanding step, does it measure the *in vivo* process it claims to represent.

Face validity refers to the extent to which a test appears to measure what it claims to measure based on face value. It would indicate that a model system recapitulates important anatomical, biochemical, neuropathological, or behavioral features of a disease or disorder. Alternatively, this could also refer to whether the model system recapitulated a normal biological/development/aging process. These two examples will depend on expectations placed on any system. Attempts to relate findings from an experimental study to a human neurological disease is difficult as there are few, if any, neurobiological abnormalities that are known with certainty to be hallmarks or biomarkers of common neurological disorders and diagnosis of any given disorder can be highly variable and inexact. Models developed by altering the expression or function of proteins, biochemical pathways, or neural connections hypothesized to be involved in the specific disease pathogenesis or that represent hallmarks of disease could be used. However, there remains a need for confirmation that these are more than interesting phenocopies. It has been suggested that, rather than attempting to model human syndromes, a more productive approach would be to define and model biological components that may account for clusters of co-varying symptoms/signs sharing common underlying neurobiological mechanisms.

Predictive validity is probably one of the more important factors for assessing the potential for adverse human health outcomes but is likely the most difficult and depends on whether one is trying to predict a very focused biological outcome or a more general health effect. Apart from targeted human studies, poisoning events, or occupational exposures, assigning causality to a specific environmental factor is difficult. Addressing causality is a primary value of *in vivo* experimental studies.

### 2.2 Reproducibility

The ability to verify experimental findings is essential and of importance if the data is to be used for a weight-of-evidence in risk assessment. Rigor, (i.e., replicability and reproducibility) can become complicated especially if examining low-level changes. Failure to replicate a specific finding can depend on the robustness of that effect. In many cases this occurs when studies are performed on small sample sizes, inadequately validated methodology, or lack of understanding of the underlying principles of the assays being performed. The ability to reproduce findings across studies or laboratories is hindered by the rarity of reports which cite details of more than a small proportion of relevant variables. In animal behavioral studies, variance can be introduced by numerous factors including stress, sex, age, and environmental factors as well as with experimental design and data analysis (Kafkafi et al., 2018; Gulinello et al., 2019). Analogous considerations apply to fields of study other than behavior and similar strategies are used to ensure reproducibility and reliability of the assay. While one normally attempts to minimize variability by placing as many factors under experimenter control as possible, the deliberate introduction of heterogeneity (heterogenization of experimental design) offers an alternative approach to improve replicability and generalizability in phenotypic outcomes (Richter, 2017; Voelkl et al., 2020; Usui et al., 2021). This approach is rarely taken in neurobehavioral assessments as the evaluation is normally based on the average group response rather than to identify variance and compare that across endpoints in individual animals to generate a pattern of effect.

## 3 Neurobehavioral Screening/Phenotyping

The regional heterogeneity of the brain with regards to the multiple neural cell populations, distinct molecular profiles, circuitry, and regional and localized specificity of vulnerability from chemicals, drugs, or genetic manipulation requires a relatively broad integrated approach to detecting neurotoxicity. For these reasons, behavioral assessments are considered as representative of the organizational neural systems inclusive of motor, sensory, cognitive, attentional, and physiological functions. They continue to be used as a surrogate index of nervous system functioning not only in experimental studies but also in the clinical setting. While appearing simple, a behavioral task that relies heavily on the execution of complex functions can offer a window into disruptions of the network integration to perform that task. For general behavioral phenotyping, behaviors have been characterized into domains of motor, sensory, learning/memory with additional consideration of social and emotional (anxiety, stress) related behaviors. The procedures to assess the multiple domains range from relatively simplistic to complex. In many cases the relative simplicity of “screening” or “phenotype” approaches, while valid, fails to address expectations of the user of such data. This may be the result of selecting testing strategies that are simple, effective, efficient, and most economical but may not be appropriate for the required assessment. One might consider that the less complicated the procedure, the easier it is to interpret the nature of the observed change however, this is not necessarily the case in that the observed behavior can be influenced by multiple factors not necessarily reflective of the behavioral domain being tested (Cory-Slechta et al., 2021). Thus, the reliance on relatively simple behavioral assessments to meet simple requirements with regards to animal use, time, and costs has come at a cost with a loss of an appreciation of the biology of the experimental organism and tailoring for the most appropriate parameters and timepoints for assessment.

Different behavioral assays are tools that can vary depending on the specific paradigm employed and the “type” of behavior evaluated. This requires knowledge of the behavior and the biological basis of that behavior for the conduct and interpretation of such studies. As an example, tests for assessing learning and memory cover multiple paradigms and they do not necessarily assess the same aspect of learning or of memory (e.g., tests for spatial memory do not measure the same thing as tests for working memory). An additional consideration is the expertise of the laboratory for conducting the test and in having a fundamental understanding of the behavior being assessed. While it is readily accepted that expertise is required for sophisticated techniques such as optogenetics, electrophysiology, image analysis, molecular analysis, the necessary expertise in behavior is often less valued but as critical.

## 4 Sources of Variability

Many types of issues related to variability are not unique to rodent neurobehavior studies but apply to many *in vivo* studies whether human, natural behavior assessments, or experimental animal. While one normally thinks about variability within a study, variability across studies and laboratories is a critical factor in the perceived validity and reliability of finding. This is often due to the lack of comparable testing paradigms and the absence of details on the testing apparatus, paradigm, and protocol within any reported study. If known and taken into consideration or placed under experimental control, one can begin to manage many potential sources of variability and their influence on data interpretation. This may require counterbalancing of multiple factors (e.g., time of day, sex, apparatus, experimenter, test sequence). When appropriate standardizations and controls are maintained the impact of experimenter and laboratory on behavioral phenotyping can be minimized (Lewejohann et al., 2006). The purpose of such steps is to account for all sources of variability to be more precise in quantifying the effects observed. In general, one would use valid test methods, have experienced investigators conduct the tests, maintain a relatively consistent environment to minimize extraneous cues and stress. By carefully accounting for and controlling extraneous sources of variability and fully reporting methodological considerations and supporting data, precision in inference can be increased and reproducibility enhanced. While these issues are often raised as being special concerns for neurobehavioral studies, they are no different than the good practices that apply to all types of experimental studies; molecular, cellular, physiological, biochemical, anatomical, or behavioral.

Additional considerations for sources of variability lie in the influence from neurological as well as non-neurological systems (e.g., hormonal, microbiome, cardiovascular) to induce or modify neurotoxicity. If one considers the complexity of behavior and its determinants, the multitude of influences on such behavior, and the individual variability of the human population, one could conclude that issues raised regarding behavioral assessments in rodents are not unlike issues with human populations. For any endpoint, multiple factors may influence the outcome and variability across studies. In addition to general biological features such as sex, strain, species, age, and health, variability can be introduced by alterations in one modality which can then affect the ability to assess other modalities (Saré et al., 2021; Vorhees and Williams, 2021). For example, deficits in motor function (levels, strength, and endurance) can contribute to longer latency measures in learning and memory task but not necessarily affect the ability of the animal to learn. Alterations in sensory capabilities such as olfactory or visual would compromise the ability of the animal to use related cues. In this case one might observe poor performance in a spatial-dependent learning task as the Morris Water Maze (MWM) that was not reflective of a learning deficit. Altered hearing can vary with strain or age and would influence auditory startle or any task employing an auditory cue. Differences in motivational levels and reinforcement value will affect exploratory and learning tasks. Reinforcement value of shock would be influenced by an animal’s pain threshold and performance on a shock-reinforced task would be altered. Elevated anxiety-state level can interfere across several behaviors.

One aspect that is not readily considered in exposure related neurological effects is the robust compensatory capability and adaptability of the nervous system. These may be represented by reactive synaptogenesis or adult neurogenesis. While serving as a prominent protective feature, but with time it can mask functional significance of damage. While basal effects may not be evident, the differences may manifest as decreased reserve capacity and ability to adapt upon future insults. These latent or underlying alterations would require some form of a challenge (physical, pharmacological, stress, disease-related) to unmask (Kraft et al., 2016).

### 4.1 Animals

There are inherent factors related to the animal under study that can influence the selection of assays and outcome (Genzel, 2021).

#### 4.1.1 Species and Strain

Multiple species display behavioral variance among individuals due to various factors (Demin et al., 2020). Within rodents, species, and strain can play a significant factor in performance on various behavioral test paradigms. This background can also influence the strength of underlying confounding factors such as response to stress. Within each rodent species, strains can demonstrate differing levels of activity, motivation, anxiety, and learning skills.

#### 4.1.2 Sex

Sexually dimorphic non-reproductive behaviors are noted in both human and animal models (Kessler et al., 2005; Beery and Zucker, 2011; Meeh et al., 2021). Sex differences are evident on several behavioral endpoints like anxiety and depression but even in assessments considered to be as simple as motor strength and coordination differences in mice have been reported due to sex and strain (Eltokhi et al., 2021). While it is recommended to include both sexes in experimental studies, how sex is integrated and counterbalanced into the experimental design depends upon the research question. This also brings forward the possibility that effects may not be evident in both sexes on the same task but could be detected with tasks optimized for each sex. In the MWM a well-known sex differences exist in that adult male rats learn all phases of the MWM test faster than females (Jonasson, 2005; Vorhees et al., 2008). In more recent studies, it was shown the sex differences in learning and memory may be due to differences in strategies and mechanisms of memories between sexes (Tronson, 2018; Chen et al., 2021) and need to be considered when choosing behavior assays and assessing the data.

#### 4.1.3 Litter Effect

The litter, defined as the maternal *in utero* environment and the post-partum environment, can have significant influence on neurobehavioral assessments. “Intra-litter likeness” (Golub and Sobin, 2020), which occurs due to genetics and shared maternal environment, can induce similar responses in littermates. Rodents from the same litter are phenotypically more like one another, as compared to offspring of another litter. This similarity contributes to a correlation in responses within litters. Such litter differences may account for a high degree of variability associated with commonly studied phenotypes (Wainwright, 1999; Lazic and Essioux, 2013; Jiménez and Zylka, 2021). In addition to the genetic and sex composition of the litter, maternal behavior, and the post-natal litter environment can significantly modify behavior (Crews et al., 2009; Sarro et al., 2014; Courtiol et al., 2018). As an example, maternal environment and sex distribution within a litter can influence some behaviors such as adolescent play behavior and exploratory behavior (Laviola and Alleva, 1995). Failure to account for litter effect can lead to reduced statistical power to detect a significant effect and increased false positive rates (Type I error), possibly masking effects of interest (Holson and Pearce, 1992; Lazic and Essioux, 2013; Aarts et al., 2014; Williams et al., 2017; Golub and Sobin, 2020; Jiménez and Zylka, 2021). To maintain some level of experimental control for post-natal litter conditions, a recommended approach is to standardize litter size and sex distribution within a few days of birth (Chahoud and Paumgartten, 2009). While of benefit, the stress of a culling procedure itself has been implicated as a confounding variable (Suvorov and Vandenberg, 2016) and thus any standardization procedure needs to be conducted in a manner to minimize stress. To further address litter effects, the litter can be considered as a unit and thus, either an average of the response of pups from each litter is considered or only one pup per sex is use for any one endpoint. Alternatively, the pups can be cross-fostering for uniform distribution of the pre- and post-natal environment.

#### 4.1.4 Age

The age of the animal is critical for determining the appropriate behavioral test and parameters for assessment. This applies not only to the maturation of the nervous system but also to the specifications of equipment. For example, hearing impairment occurs with age especially in mice thus, shifting the auditory stimuli to an air-puff stimuli for startle response assessment would be an option (Shoji and Miyakawa, 2018). Assessment of motor activity of young animals within a photocell arena requires that the distance between the photocells is appropriate to capture a sufficient number of events for valid analysis. A similar issue would arise for rearing behavior if the photocell bank was not empirically determined for age and size of animal to accurately capture hindlimb rearing rather than simply top of head or top of back. Similar issues arise for determining age-specific physical characteristics of any test apparatus.

Maturation of the nervous system circuitry is also a critical factor in that many assessments require stages of development for valid assessment. There is a wealth of data on the neural circuitry involved with acoustic startle response (ASR) and prepulse inhibition (PPI) and with the developmental ontogeny of the response and the corresponding network formation (Shnerson and Willott, 1980; Yeomans and Frankland, 1995; Swerdlow et al., 2001; Swerdlow et al., 2008). The acquisition and retention of a passive avoidance response is a function of age with rats younger than 28 days of age showing less response strength and retention (Schulenburg et al., 1971). Maturation of the prefrontal cortex, amygdala, and striatum is necessary for the expression of active avoidance requiring inhibitory signaling in the rodent prelimbic prefrontal cortex (Jiao et al., 2015; Diehl et al., 2019). Animal age can also influence MWM performance not only the strength effect on latency but also in the type of learning. Comparing the ontogeny of allocentric learning, cued learning but not spatial learning can be evident as early as postnatal day 17. Spatial learning requires further maturation to postnatal day 23 (Tonkiss et al., 1992) or 28 (Schenk, 1985). Further work demonstrated that very young rats (PND17-19) can learn the spatial aspect of the MWM however, this required additional of extra cues (Carman and Mactutus, 2002).

### 4.2 Housing/Environment

Variables in housing condition (e.g., single vs. group, cage material, ventilation, bedding), have health and neurological effects that can alter rodent behavior (Burn et al., 2006; Castelhano-Carlos and Baumans, 2009; Åhlgren and Voikar, 2019). On a simple note, cage size and animal density can significantly alter activity levels in mice (Poon et al., 1997). This may be the result of a learned response due to space restrictions, social dynamics, or a combination (Cavanagh et al., 2011; Duan et al., 2021). Social interactions of play and other behaviors in weanlings and across the lifespan influence neurobiological mechanisms affecting behavior. For example, socially isolating animals by singly housing induces physiologic abnormalities and alters motor, memory, and social tasks (Ferrari et al., 1998; Võikar et al., 2005; Kercmar et al., 2011; Kulesskaya et al., 2011; Kamakura et al., 2016; Saré et al., 2021). While group housing is recommended, it can lead to social hierarchies and inter-male aggression (Hånell and Marklund, 2014). Recent work from Duan et al. (2021) showed an increase in anxiety-like behaviors in a mouse model of chronic social defeat stress where an aggressive mouse was allowed to bully another mouse for a few minutes per day and allowed full day smell and sight interaction. This effect was not limited to behavior but was associated with an increase in mitophagy and decrease in mitochondria in neurons of the amygdala. Locomotor activity and learning and memory can also differ between dominant and subordinate animals (Dubrovina et al., 1997; Ferrari et al., 1998) and the reaction to social stress can alter reward-based learning (Cavanagh et al., 2011). Thus, while in a large animal study the housing considerations are often dictated by logistics, an understanding and appreciation of the impact of different housing conditions remains of importance.

The physical features of housing can affect behavior. The issue of foot lesions, altered skeletal muscle, and balance observed in wire-bottomed cages is evident in behavioral tests such as rearing, rotarod, and grip strength. It has been reported that housing in wire-bottom cages resulted in higher corticosterone levels in F344BNF1 rats following acute restraint stress (Freed et al., 2008). The shift to plastic, solid bottom home cages has addressed these issues (Manser et al., 1995; Miller et al., 2020). The open-top cage microenvironment is directly influenced by the animal room environment and rodents are stimulated with pheromones emitted by their congeners in the same room. In individually ventilated cages, rodents are olfactory isolated, creating an issue for puberty onset and estrous cyclicity presenting confounding issues for studies examining changes in behavior due to endocrine function (Sales et al., 1988; Turner et al., 2005). In these cages, or in inhalation cages, electronic equipment or high ventilation rates can increase noise exposure and heat loss affecting behavioral performance (Baumans et al., 2002; York et al., 2012; Polissidis et al., 2017). Environmental enrichment (e.g., cage sizes, natural bedding, nesting material, shelters, and toys) (Sparling et al., 2010; Girbovan and Plamondon, 2013) is now a general requirement for animal studies however, the type of environmental enrichment can influence behavior (Boehm et al., 1996; Soffié et al., 1999).

### 4.3 Animal/Experimenter Interactions

All behavioral tests require interaction between the animal and experimenter. While the experimenter (e.g., personal features or level of training) can influence outcomes, rodents can also differ in their response to humans (Schallert et al., 2003; Hurst and West, 2010). Handling prior to behavioral testing allows the animal an opportunity to adapt to the experimenter and can reduce stress-related effects and outcome variability (Schmitt and Hiemke, 1998; Hurst and West, 2010). The lack of adaptation to handling before the start of a study may result in an alteration of the response over time due to the adaptation to human contact. Alternatively, depending on the research question and the type of behavior assessed (e.g., stress response), one might want to consider the use of relatively handling-naïve animals. This could be considered in assays assessing anxiety-like behaviors or social interactions but also in any assay assessing exploratory activity. In addition to general handling, the time of handling prior to or within a test can present as a variable (Lorenzini et al., 1990; Schmitt and Hiemke, 1998; Gouveia and Hurst, 2017).

### 4.4 Experimental Conditions

#### 4.4.1 Quality of the Experimental Manipulation

One of the more challenging aspects of examining the effect of any experimental manipulation is to ensure the “purity or accuracy” of your manipulation (e.g., genetic background, genetic manipulation, uniformity of the experimental manipulation, pharmaceutical or chemical purity, chemical stability). With air pollution and nanoscaled chemicals, additional parameters of physical characteristics require consideration (Bencsik and Lestaevel, 2021; US EPA, 2022). For neurotoxicity, the additional question arises of target tissue exposure estimates (Rees et al., 1990).

#### 4.4.2 Physical Factors

Time of day and lighting conditions can influence performance (Richetto et al., 2019). The lighting conditions should be consistent with the research question and clearly stated. It is normally considered that since rodents are nocturnal that activity measures would be more sensitive if conducted either during the dark cycle or under red-light conditions. However, what is important is a coherent choice with several tests done during the same phase. For example, for reproductive behaviors it is important to do these tests together with anxiety and locomotor activity during the dark phase. For tests of learning and memory, together with anxiety, activity, startle, the norm is to conduct test during the light phase. If avoidance of an open area is to be examined, then a well-lighted environment should be used to strengthen the negative reinforcing properties brightly light center of the arena. Environmental temperature would be of additional importance in animals with deficits in thermoregulatory control (Lindner and Gribkoff, 1991).

#### 4.4.3 Acclimation

Acclimation to a testing environment is dependent upon the behavioral paradigm. It can be of importance in tests of motor strength/coordination or learning and memory. Stress-related hormones such as corticosterone, vasopressin, oxytocin, and adrenocorticotropic hormone are often altered unless the animal has adapted to the testing environment (Coover et al., 1986; De Boer et al., 1990; Engelmann et al., 2006). While this would be an accurate read-out of an effect upon performance, in the absence of adaptation it would remain a question as to whether this represented a deficit in learning/memory. In comparison, if the novelty of the environment is a factor, then naïve animals would be preferred as prior acclimation interferes with the paradigm.

#### 4.4.4 Behavioral History

In most behavioral phenotyping or toxicity studies, any one animal cohort is subjected to multiple behavioral tests. While this is a cost and effective approach, the influence of behavioral testing on subsequent behavioral assessments or responses to pharmacological agents requires consideration (Barrett, 1977; Holmes et al., 2001; McIlwain et al., 2001; Blokland et al., 2012; Von Kortzfleisch et al., 2019). Prior test experience and order effects have been observed in open field, rotarod, hot plate, and forced swim. Prior exposure to the test apparatus can alter exploratory behavior and activity as the novelty of the environment is diminished. Painful experiences such as hot-plate or foot-shock can modify performance on subsequent tests that may be related to stress-related hormonal changes such as elevated corticosterone (Daviu et al., 2010). Additionally, unexpected impacts on subsequent behavior can occur from the learned aspects of negative stimuli (Daviu et al., 2010). Similar types of behavioral impact can occur with negative contrast effects that occur following negative emotional responses such as frustration associated with increasing complexity of a response schedule or with a reward down-shift (Flaherty, 1996). It has been suggested that an individual response to stress may influence a response to reinforcement. As an example, stress-induced elevations of cortisol and dopamine levels have been implicated in activating the brain’s reward system (Campioni et al., 2009).

Unintended behavioral histories based on home-cage environment (e.g., social hierarchy) can influence outcomes. Additionally, behavioral experience, in and of itself, can modify the brain with regards to molecular, biochemical, and behavioral performance (Bennett et al., 1964; Ferchmin and Eterović, 1986; Wallace et al., 1992; Mohammed et al., 2002; Kozorovitskiy et al., 2005; Rosenzweig, 2007; Geng et al., 2021). These influences are demonstrated with experience driven synapse formation and regulation. As an example, sensory of motor activity induced by spontaneous motor activity in the early stage of development is sufficient to self-organize spinal reflexes (Marques et al., 2014) and is instrumental for coordination of activity in sensorimotor spinal cord circuits (Inácio et al., 2016). Behavioral experience continues to modify the brain throughout adulthood which serves as a basis for facilitating functional recovery after brain damage with development of compensatory behavioral strategies and neuronal restructuring (Jones et al., 2003).

## 5 Behavioral Assessments

Behavioral assessments overall cluster into categories of standard scoring methods. There are methods that require observer scoring which are potentially subject to unconscious rater bias and semi-automated or fully-automated systems that minimize issues of observer bias. However, automatic data collection often requires observation confirmation of any specific outcomes for interpretation (e.g., stereotypic behavior, freezing behavior). Recent efforts to use fully automated assays that generate large and complex data sets such as, automated home-cage monitoring or machine learning approaches, involve extensive data acquisition of multiple endpoints that may introduce ambiguities that limit the ability to interpret biologically relevant alterations in the absence of a targeted experimental question/design (Robinson and Riedel, 2014; Wiltschko et al., 2015; Mingrone et al., 2020).

### 5.1 Observational Batteries

Early work in “screening/phenotyping” took an approach to focus evaluations based upon assessments conducted in the clinical diagnostic arena (Goldberg, 2017) which are represented in recommendations to the World Health Organization neurobehavioral core test battery for human assessment (Anger, 2014). Thus, a rationale was built for the inclusion of observational batteries to assess rodent sensory, motor, and autonomic system functions with additional assessments of learning and memory (IPCS, 1986; IPCS, 2001).

Like human neurological exams, rodent observational batteries often serve as a first-tier test to identify “an effect.” Examples include the Functional Observational Battery (US EPA, 1994b; Moser and Kallman, 2018; Gauvin, 2021) and the SmithKline, Harwell, Imperial College, Royal Hospital, Phenotype Assessment (SHIRPA) (Rogers et al., 2001; Lalonde et al., 2021). Additional batteries have been developed for assessing vestibular dysfunction (Maroto et al., 2021), various genetic manipulations (Brown et al., 2000), and neonatal function (Fox, 1965; Brown et al., 2000; Feather-Schussler and Ferguson, 2016; Soria-Ortiz et al., 2021).

### 5.2 Motor Assessments

Motor assessments serve multiple functions within a phenotyping battery in that they allow for interactions between animal and experimenter, assessments of general health indicators, body posture, stance, and identification of functional alterations that may compromise interpretation of a nervous system specific effect in subsequent studies. Motor assessments can be conducted either by observational methods or by semi-automated approaches. In general, behavioral phenotyping relies heavily on assessing motor activity within an arena (see below) however, assessments using home-cage running wheels have also been used (Novak et al., 2012; Chomiak et al., 2016; McPherson et al., 2018; Zhu et al., 2021).

If deficits in function or limb strength are considered then one may include assessments of gait, grip strength, or motor coordination. While grip strength can be relatively easily assessed using a strain-gauge it relies heavily on the experimenter. Gait analysis, motor coordination, and skilled reaching task are not normally a single assessment but require some level of animal training and repeated testing to obtain accurate assessments (Montoya et al., 1991; Yu et al., 2010; Alstermark and Pettersson, 2014). Each of these approaches can have as aspect of learning in the test paradigm. Bilateral loss of vestibular function produces a syndrome of abnormalities in motor behavior characterized as hyperactivity, stereotyped circling, backward displacement, and abnormal head movements and while electrophysiological methods and test batteries are available for evaluation, they are rarely conducted (Llorens and Rodríguez-Farré, 1997; Llorens et al., 2018).

#### 5.2.1 Motor Activity and Open-Field Test

As a general default, neurotoxicity testing or behavioral phenotyping utilize automated photocell devices to record motor activity. Alternatives include the use of a home cage running wheel or implanted microchips to monitor activity levels and circadian cycle-related activity. The original published open-field apparatus for rats consisted of a circular arena (to eliminate corners) of approximately 1.2 m in diameter enclosed by a high wall (Hall, 1934). As originally designed, the procedure exposed the rodent to an adverse environment (bright light) from which they could not escape thus, including an aspect of “emotionality” in the assessment. An arena type assessment of motor activity includes an aspect of exploratory behavior as well as general motor function, various diversified paradigms have developed over time targeted to the aspect of behavior under study (Walsh and Cummins, 1976; Prut and Belzung, 2003; Lipkind et al., 2004; Seibenhener and Wooten, 2015).

Automated photocell and video capture detection systems allow for the assessment of multiple aspects of motor and exploratory behavior. Using the stimulus of novelty, free-exploration in the arena can be used to examine curiosity and exploration (Pisula and Modlinska, 2020). Modifications to the arena recording configuration can allow for additional assessments as relevant to the research question. For example, measures related to fear behavior can be monitored as thigmotaxis (wall hugging) or resistance to enter the center of the lighted arena. A general pattern of activity will show elevated levels in the early epochs, decreasing over time as the animal learns and acclimates to the test apparatus. The acclimation pattern is normally examined over epochs. Any data analysis and interpretation requires consideration of interdependency and behavioral competition within the assay (Frussa-Filho et al., 2016). For example, ambulation will proportionally decrease if rearing increases or if stationary grooming increases. While fine movements of grooming and stereotypic behavior can be captured with many automated systems, caution is encouraged as they require observational confirmation of any specific behavior (grooming, stereotypic behavior). With photocell devices and video imaging software, the availability of heat-map tracing of ambulatory activity allows for patterns of activity and exploration to be examined. In a recent review, Thompson et al. (2018) discussed aspects of the neurobiological basis of exploration and the identification and quantitation of organized movement subsystems in rodent that allows for a more detailed analysis of the pattern of exploration.

Open-field behavior can be modified by a variety of factors, such as size and shape (circular, square, rectangular) of the arena, the zone configuration of the arena, lighting conditions, light/dark cycle, duration of the testing (e.g., a 10 min session will not provide acclimation data and, unless compared to an exact time interval, cannot be compared to a longer 30–45 min session), animal housing conditions before testing (social, individual), diet, species, strain, sex (adult females often more active than males), and age (decreased activity with aging; photocell placement appropriate for size of animal), familiarity with the apparatus (single exposure, repeated testing) (Walsh and Cummins, 1976; Choleris et al., 2001; Tou and Wade, 2002; Eilam, 2003; Prut and Belzung, 2003; Lipkind et al., 2004). As an example, assessments under red-light will provide a different distribution of activity as compared with assessments under dim-light or under the normal lighting conditions. Additional factors for consideration are related to the investigator’s selected or software algorithms used for calculating specific endpoints such as 1) ambulatory activity (a simple addition of counts of the x and y plane or an adjustment to capture the tangential movement of the animal), 2) total activity (e.g., ambulatory activity + fine activity + rearing as compared to ambulatory activity + rearing). One additionally component that can introduce variability across studies is the requirement to empirically determine criteria for rearing (e.g., setting photocell bank at a size appropriate height to capture only rearing). Measuring ambulatory activity of very young rodents using photocell devices requires consideration of the photocell placement to capture a sufficient number of events for analysis.

### 5.3 Sensory Responses

Alterations in sensory processes (e.g., paranesthesia, auditory, visual, or olfactory) are often reported symptoms in humans with toxicant exposure or in neurological disorders. Screening procedures have been devised to detect overt sensory deficit or dysfunction in animal models not only for direct effects but also for the impact on subsequent behavioral performances.

#### 5.3.1 Olfaction

The olfactory system is critical for multiple aspects of behavior in the rodent (Grabe and Sachse, 2018; Dorman and Foster, 2021). The main olfactory system starts in the olfactory epithelium of the nasal cavity containing sensory olfactory neurons, glial-like sustentacular cells, and basal cells. In humans, olfactory dysfunction takes multiple forms including decreased (i.e., hyposmic) or loss (i.e., anosmic) ability to detect or correctly label odors. Dysfunction has been observed in animals (Coronas-Sámano et al., 2014; Bermúdez et al., 2019) and in humans associated with viral infections, idiopathic Parkinson’s disease, Alzheimer’s type dementia (Boesveldt et al., 2017; Doty, 2018; Genter and Doty, 2019) and with exposure to ∼200 toxicological compounds (Dorman, 2018; Genter and Doty, 2019). Most assessment methods can be conducted by simple tests as within an observational battery, preference response to home-cage odors, innate responses to predator odors, recovery of hidden food or olfactory habituation/dishabituation tests, and olfactory discrimination tests.

#### 5.3.2 Pain

The sensation of pain is a result of central nervous system processing and is not directly measured in rodents. Rather, nociception is used to describe the peripheral neuronal response to noxious stimuli, which can be mechanical, thermal, electrical, or chemical (Dubin and Patapoutian, 2010). Behavioral methods used to quantify and evaluate pain-like behaviors (withdrawals) in non-anesthetized animals are categorized as stimulus-evoked or non-stimulus methods. Examples of stimulus methods include von Frey, Randall-Selitto (paw pressure), heat stimuli (hot plate, tail flick, Hargreaves test), and cold stimuli (cold plate, acetone, cold plantar assay). Non-stimulus evoked methods such as burrowing, weight bearing, and gait analysis can be used to evaluate spontaneous pain (Tappe-Theodor and Kuner, 2014). The neural circuitry underlying response to noxious stimuli overlaps with reward circuits and circuits important for cognition. While standard behavioral phenotyping or neurotoxicity testing often does not systematically assess pain threshold, pain modifies an animal’s motor activity, learning and memory, and decision making (Low, 2013; Neugebauer, 2015; Watanabe and Narita, 2018). If painful stimuli are used as a reinforcer, reinforcement value can be altered with changes in pain threshold.

#### 5.3.3 Startle and Prepulse Startle Inhibition

The startle response is an unconditional reflex, characterized by the rapid contraction of skeletal muscles, in response to a sudden and intense startling stimulus (e.g., noise burst, air puff, light flash). It shows consistency across species, represents a relatively simple neural circuitry, and is sensitive to a variety of experimental manipulations. In rodents, the acoustic startle response (ASR) can be used to study habituation, sensitization, classical conditioning, fear, and anxiety. Habituation to the startle response is a form of non-associative learning and can also be viewed as a sensory filtering process as it decreases an organisms’ response to a non-threatening stimulus. Habituation can be examined within a test session (short term habituation) or across sessions (long-term habituation). Within a session, habituation normally occurs within the first 10 trials and over 4–5 days for across sessions (Valsamis and Schmid, 2011; Pilz et al., 2014). Prepulse startle inhibition (PPI) describes the phenomenon in which a weak initial stimulus (prepulse) inhibits the startle response that is elicited by a strong stimulus. Recent articles on procedural methods and optimization considerations for ASR and PPI are available (Valsamis and Schmid, 2011; Shoji and Miyakawa, 2018; Hormigo et al., 2019; Miller et al., 2021). The primary data collected is on the strength of the reflex response, therefore attention is required for optimizing the system (restraint and sensitivity) for the age and weight of the animal. In addition to strength of the response, the time to respond is recorded. This is a variable that rarely differs across animals in a study and thus, if observed one needs to reconsider the test protocol.

PPI is generally considered a behavioral response to changes in an acoustic or multimodal stimulus input that may allow for assessment of effects on brainstem and higher order processing (Swerdlow et al., 2016; Gómez-Nieto et al., 2020). The pathways involved in sensory processing of a given pre-stimulus depend on stimulus parameters such as frequency spectrum, amplitude modulation, intensity, and interval between pre-stimulus and startle-eliciting stimulus. Changes in PPI have been linked to various neuropsychiatric disorders with the strongest association demonstrated for schizophrenia, obsessive compulsive disorder, and Tourette’s syndrome and those seen with autism-spectrum, attention deficit hyperactivity, or post-traumatic stress disorders are not as clearly defined (Braff et al., 2001; Swerdlow et al., 2008; Li et al., 2009; Kohl et al., 2013; Mena et al., 2016). As such, PPI has been promoted as a potential biomarker of brain function in the context of disease and allows for translation across species.

Startle and PPI regulatory circuitry (i.e., the forebrain circuits that descend to regulate the primary pontine startle and PPI mechanisms) develop into adolescence (Shnerson and Willott, 1980; Yeomans and Frankland, 1995; Swerdlow et al., 2001; Swerdlow et al., 2008). Thus, development of the primary circuitry and the descending regulatory circuits are critical issues for interpreting data across early ages.

While commercially available equipment allows for an ease of conducting these types of experiments, reproducibility, and data interpretation require optimization and standardization of the test paradigm. Multiple protocols and review are available (Reijmers and Peeters, 1994; Geyer and Swerdlow, 2001; Valsamis and Schmid, 2011; Lauer et al., 2017; Miller et al., 2021) but are at the discretion of the investigator and a source of variability. As numerous variables can contribute to the data outcome, efforts to optimize parameters should be conducted prior to any study. These include confirmation of optimal startle intensities (prepulse and startle) in naïve animals for specific test ages, species, and strain. This involves an input/output function test sampling across startle stimuli intensities to determine the maximum startle response and to average the response over the entire response window. Prepulse stimulus intensities should be sub-threshold and elicit intermediate levels of PPI to allow for detection of treatment-induced increases or decreases. Settings for maximum startle response to each individual prepulse intensities and the required number of intervals for PPI intensities should be confirmed on a regular basis to maintain uniformity across studies and to confirm no experimental shift in their sub-threshold nature. While pre-pulse intensities may be set for a specific species, strain, or age confirmation of the sub-threshold status in experimental animals is required for interpretation. If shifted, this would reflect in the PPI responses. It is recommended that response to each individual pre-pulse intensity be examined prior to initiating a full PPI schedule. Optimally, this should be conducted a few days before the PPI but could be triggered upon the PPI results and conducted within a short time interval. Testing conditions and schedule reporting should include information on period of acclimation to the holder, background noise level (which can drift over age of equipment), sampling window, the intertrial interval, prepulse stimuli intensities including duration and inter-stimulus interval prior to onset of startle stimulus, and the testing delivery schedule of stimuli (Geyer and Swerdlow, 2001; NTP, 2015). These may vary depending on equipment, sex, species, and strain of animal as well as the hypothesis under study. The sampling window is a parameter that can be modified based on the research question and can be a prominent source of variance across studies and laboratories. While this is a general practice for rodents, similar issues with regards to individuality, sources of variation, and stimulus-experience history have been reported for SR and PPI in zebrafish (Medan and Preuss, 2014; Pantoja et al., 2016; Kirshenbaum et al., 2019; Beppi et al., 2021).

Data compilation and analysis represent areas for variability across studies. This can be dependent somewhat on the testing schedule and determination of PPI denominator (Csomor et al., 2008). The impact of stimuli history suggests that the true reflex startle response is limited to the first response within a testing paradigm. This is followed by reflex habituation to the stimuli, normally observed across the first 10 stimuli presentations. A general PPI testing paradigm normally presents a series of initial 120 dB trials followed by blocks of trials that are representative of PPI/120 dB pairing and 120 dB responses. A testing schedule can either include a series of 120 dB trial preceding these “blocks” or directly initiate the PPI testing schedule. If initiated immediately, the shifting response to 120 dB due to habituation needs to be taken into consideration. Extending the session to include excess pairings can be confounded by a reflex adaptation with multiple stimuli deliveries that diminishes the signal for determining inhibition.

### 5.4 Learning and Memory

An organism is required to employ different strategies of learning to become proficient in any task. Learning paradigms require analysis of performance over trials to demonstrate acquisition. There are multiple paradigms to assess learning and memory however, they do not uniformly evaluate the same type of learning and thus, are not interchangeable. Memories can be classified according to different criteria based on function (working versus reference); content (declarative/explicit versus procedural/implicit); duration (e.g., immediate, or short-term versus long-term or remote); nature (associative vs. non-associative); or motivation (appetitive/reward vs. aversive) (see Quillfeldt, 2016).

#### 5.4.1 Mazes

Open mazes primarily use environmental visual/spatial cues to measure place learning and memory. These different maze paradigms are not identical but rather examine different processes that contribute to or affect spatial learning (Vorhees and Williams, 2014a). They vary in apparatus configuration, availability of visual/spatial, associative, or sensory cues; complexity of the task, and motivation driver (escape or food). Therefore, “spatial” abilities measured in one procedure may not resemble those engaged in another but rather depends on the learning paradigm employed. In evaluating spatial learning and memory, paradigms have employed the radial arm maze test, T and Y mazes using spontaneous alternation and win-shift tests, Morris water maze, Barnes maze, Cincinnati maze, and spatial paradigms in the novel object recognition test (Hodges, 1996; Paul et al., 2009; Morellini, 2013; Rosenfeld and Ferguson, 2014; Levin, 2015; Mohseni et al., 2020). Each can be modified to address specific questions with regards to learning and memory not only in the aspects of the learning paradigm but with subsequent assessments to assess memory and cognitive flexibility. Working memory corresponds to a critical cognitive domain required for the representation of objects or places during goal directed behavior. Reference memory is required for temporally stable representations of those objects or places. Various protocols for each are available (Dudchenko 2004; Vorhees and Williams, 2014a; Hussein et al., 2018).

#### 5.4.2 Morris Water Maze

The Morris Water Maze (MWM) is a common method used for assessing rodent spatial learning and memory (Morris, 1984; Vorhees and Williams, 2006; Terry, 2009; Vorhees and Williams, 2014b). It relies on a natural behavior of the animal with an equal level of motivation over a wide range of physiological conditions. It requires minimal training, and, with rare exceptions, all experimental animals successfully perform the task in a relatively short period of time. While appearing simple, it is a challenging task that employs a variety of sophisticated mnemonic processes, e.g., acquisition and spatial localization of relevant visual cues that are subsequently processed, consolidated, retained, and then retrieved for successfull navigation to locate a hidden platform for escape (Morris, 1984; McNamara and Skelton, 1993). It is considered that the general processes used for “visuospatial navigation” in rats also contribute to human cognitive processes.

The general paradigm allows the animal to navigate a circular water-filled tank to find a hidden platform for escape. Considerations related to maze configuration, platform location, and platform zone are discussed in various protocols and reviews (Vorhees and Williams, 2006; Terry, 2009; Vorhees and Williams, 2014b). The animal accomplishes this task using visual cues within the spatial environment. The MWM paradigm is adaptable, and variations can be employed. Versions of the MWM have been developed to assess working memory and discrimination (Morris, 1984; Stewart and Morris, 1993), distinguish hippocampally-mediated from striatal-mediated learning (Packard and McGaugh, 1992), and valuate preference of place from directional navigation (Hamilton et al., 2009).

##### 5.4.2.1 Visual and Hidden Platform Test

As performance in the MWM is latency dependent, the first step is to confirm the ability of the animal to perform the task due to physiological features (motor strength, vision, stress response). The task usually consists of ∼3 days of multiple trials with the platform visible and no external visual cues to eliminate a “spatial” aspect. This is followed by several sequential daily training sessions requiring the animal to use visual spatial cues to identify the hidden platform location. Multiple trials per day over multiple days are run to reach a predetermined criterion of learning. Learning is demonstrated not by latency to reach the platform on the last training trial but rather by acquisition, i.e., an increase in performance over training sessions. Thus, for the visual platform and the hidden platform tasks the appropriate data analysis is to calculate the average latency over any 1 day and then use a repeated measures analysis of variance (RM ANOVA) to demonstrate any differences in acquisition within each task. Consideration of latency on the first training session can provide some insight into motor effects if the animals are not beginning at the same starting point.

##### 5.4.2.2 Probe Test

Once learning criteria have been met, memory is assessed by a Probe Test. To ensure that the probe test reflects consolidated reference memory, a 24-h interval from the platform training is included (Baldi et al., 2005). The determining factor is to demonstrate a preference for the area (quadrant) in which the escape platform had been previously located (goal quadrant) as demonstrated by time spent, distance travelled, and number of entries in goal quadrant relative to other quadrants. Additional measures can be taken of latency and distance traveled to the first crossing into the goal quadrant. Given the small size of the platform and thus, the small number of observations, a general recommendation is to increase the platform zone area rather than use the actual platform size for recording the number of times the animal crosses [see (Vorhees and Williams, 2006)]. Once the platform is not found in the original site, the animal shifts its search strategy to other escape locations, raising the possibility of extinction of the behavior. Limiting probe trials to no longer than 60 s minimizes extinction and examining behavior in 30 s epochs provides a way to capture the exploratory shift (Blokland et al., 2004; Vorhees and Williams, 2014a; McPherson et al., 2018). The acquisition and probe test performances are associated with hippocampus function (Barnes, 1979; Kennard and Woodruff-Pak, 2011; Negrón-Oyarzo et al., 2018).

##### 5.4.2.3 Reversal Learning

While many MWM experiments in the literature terminate assessment after the probe test, extending the procedure to include “reversal-learning” trials offers significantly greater sensitivity. In this task, the escape platform is moved to a new location and a hidden platform task with identical visual cues to the original task is conducted. As the animal has already learned the physical requirements to perform the task, this reversal learning results in shorter initial latencies and normally requires only 3 sessions to show the shift. The inability to shift to a new escape platform location is considered to reflect a deficit in cognitive flexibility (Latif-Hernandez et al., 2016; Nolan and Lugo, 2018; Shah et al., 2018) and adaptability to changed contingencies (Whishaw and Tomie, 1997). The trials are considered reflective of frontal cortex function (Crews and Boettiger, 2009; Chawla et al., 2017). Inclusion of a subsequent probe test is an option.

##### 5.4.2.4 Swimming Speed

While swimming speed can be calculated at different times during the session, determination of the appropriate interval can influence the outcome. For example, differences on the first few trials may represent response to a novel environment, differences during the last trial of a day may reflect fatigue, and during a later session may reflect a difference in the aversive level of the water. In the probe trial, swimming speed can change of the trial with the addition of frustration of not finding an escape. Age can also impact swim speed in that latency is usually longer in adolescent mice than in adults, but distance travelled may be equivalent. Similar differences may occur in females as compared to males of the same age.

##### 5.4.2.5 Modifying Factors

Extraneous modifying factors for MWM include housing condition, handling, and room and apparatus cues (Hamilton et al., 2007). While not specific for MWM, learning is enhanced by acclimation of handling (Hölscher, 1999), home-cage environmental enrichment (Tees et al., 1990; Vorhees et al., 2008), and group housing (Wade and Maier, 1986). Performance can be influenced, especially in the probe test, by olfactory cues (urine) left by the previous animal if the water is not disturbed between animals. Of most importance is the impact of the visual cues and the size of the tank and size and location of platform.

#### 5.4.3 Radial Arm Maze

The radial arm maze (RAM) is an 8-arm maze that can be used either dry (e.g., food reinforcement) or submerged in water [e.g., escape from water (Penley et al., 2013; Macheda et al., 2020)] to assess spatial working memory and reference memory. Various procedures exist using the RAM. Paradigms can include endpoints that are latency dependent or “arm choice” dependent. Each of these paradigms can be modified to increase the complexity of the task and to measure different aspects of learning/memory (Sage and Knowlton, 2000; Levin, 2015; De Luca et al., 2016). Ability of the subject/animal to learn an effective strategy to complete the task is examined, choice behavior. One of the more common paradigms for the dry RAM is the win-shift task where the animal learns to retrieve food at the end of each arm following a pattern of not entering a previously reinforced arm thus, there is a shift to a new arm after reinforcement from a different arm. As reinforced arms diminish over the session the level of difficulty rises. Working and reference memory can be distinguished in the RAM. For this, only some of the arms are baited while others are never baited. The first entry into the baited arms is reinforced but not subsequent entries or entries into never-baited arms. Working memory is assessed by re-entries into formerly baited arms and a test for reference memory is determined by any entry into a never-baited arms. The complexity of the task can be modified with the baiting of arms of the maze and the task requirements and can include a delay or a delayed matched to sample paradigm. Non-spatial memory can be assessed by pairing reinforcement with visual or textual cues. In general, one records latency to enter first arm, latency to complete task, number of errors (entries into non-reinforced or previously entered arms) and evaluates acquisition.

#### 5.4.4 Barnes Maze

The Barnes maze (BM) assesses spatial working memory, spatial reference memory (short and/or long term), and cognitive flexibility by relying on an animal’s aversion to open spaces (Barnes, 1979; Rosenfeld and Ferguson, 2014). The BM apparatus is a raised circular platform consisting of ∼18 circular holes evenly spaced around the periphery with an escape box located under one peripheral hole. Mildly aversive stimuli (e.g., bright overhead lights) provide motivation to locate the escape box. Visual cues are provided for spatial orientation (O'Leary and Brown, 2013). Latency and distance travelled to the escape box are recorded over multiple trials across multiple sessions for acquisition and in reversal learning. Memory can be assessed in a probe test with demonstrated preference for the escape quadrant (Paul et al., 2009; Rosenfeld and Ferguson, 2014). Type of search strategy (i.e., random, serial, or direct) can be categorized. The protocol can be customized to specific research questions with maze size, number of holes, light intensity, inclusion of habituation trials, and duration of the training trials, and number of trials/session and number of sessions influencing the outcome (O’Leary and Brown, 2012; Gawel et al., 2016; Gawel et al., 2019).

#### 5.4.5 T-Maze

The T-maze is an apparatus that can be used to evaluate exploratory activity, learning, and memory (Deacon and Rawlins, 2006). Typically, either a spontaneous alternation paradigm or a reinforced alternation paradigm (working memory) is used. Naïve rodents display a tendency to alternate their choice of entry into an arm of the maze (e.g., an entry into the right arm on trial 1 is likely to be followed by an entry into the left arm). The normal spontaneous alteration pattern is examined over trials in quick succession as a measure that the animal remembers the arm visited in the previous session. With the inclusion of reinforcement, one arm is considered the “correct” arm. Training continues until the animal reaches a previously defined performance criterion of number of correct choices and latency. A multiple T-maze test is a complex maze with multiple T-junctions used to address questions of place vs. response learning and cognitive maps. Performance is measured as a right or wrong choice at each T-intersection. A rodent is placed in the maze and allowed to explore freely for a defined period across a few trials. This can then be modified to examine performance under reinforcement conditions. Acquisition is measured as a decrease in latency and a decrease in incorrect choices at junctions. Modifications to the testing paradigm can allow for the assessment of different aspects of learning and complexity (Conde et al., 1999; Locchi et al., 2007; Edsall et al., 2017). A variation on the this is the Cincinnati Maze which is an asymmetric multiple-T maze arranged in a manner that rats are required to find path openings along the walls of the maze rather than the ends to reach the goal (Vorhees and Williams, 2016). The intent is to assess the ability to use self-movement and internal cues for egocentric navigation. This involves circuitry in the dorsal striatum and connected structures. This is in contrast with allocentric navigation where external cues are employed and involves the hippocampus, entorhinal cortex, and associated networks.

#### 5.4.6 Y-Maze

The Y-maze is an apparatus with three arms oriented at 120-degree angles from each other that can be used to assess exploratory behavior (animals tend to enter a less recently visited arm) or, in conjunction with cued reinforcement, to examine learning and memory for arm selection. A Y-maze paradigm can record spontaneous alternation of arm entered or arms entered for reinforcement. Over trials, the sequence of choices is recorded to identify the pattern of alternation from one arm to the other. Acquisition is represented in the decrease in the number of choice errors (entering the incorrect arm). Latency to the goal box or choice point can be recorded. Testing paradigms can be designed to assess working memory and spatial memory (Kraeuter et al., 2019).

### 5.5 Schedule Controlled Behavior

Schedule-controlled behavior offers additional options to the investigator to work with the behavior of an animal to place it under some level of parameter controls and to increase complexity and demand. While it offers a very powerful tool to assess learning capability and limitation, it is rarely used in assessments of neurotoxicity, given the demands of time, expertise, and specialized equipment. There are however, numerous investigators who have integrated this approach into neurotoxicity assessment (Harry and Tilson, 1982; Moser et al., 1987; Brockel and Cory-Slechta, 1998; David et al., 1998; Morris-Schaffer et al., 2019). These procedures normally fall under the operant conditioning process in which new behaviors are acquired and modified through their association with consequences. While reinforcement schedules can take place in naturally occurring learning situation, the primary paradigms in neurotoxicity assessment utilized operant chambers to facilitate the schedule of reinforcement and capture behavior. Multiple test paradigms employing appetitive or aversive stimuli allow for increasing complexity to evaluate the nature of a learning deficit. In a more standard paradigm, animals are placed within an operant chamber containing a bar that can be pressed or an opening for a nose poke to deliver a food pellet or fluid reward; the animals then are “shaped” to perform the operant task (bar press, nose poke) on a continuous reinforcement schedule during which every appropriate behavior result in reinforcement. Once the animal is trained, the paradigm can shift to a different schedule of reinforcement. This different schedule can enable examination of the animal’s ability to learn a temporal association with behavior and reinforcement (interval) or an association of number of behavioral events and reinforcement (ratio), each of which can be set as fixed or variable. These can increase in complexity or demand on the animal. Additional test paradigms and schedules can be used to test for other aspects of learning including various modifications of cues, time delays, and reinforcement value as well as behavioral extinction. Recent semi-automated home-cage systems including some aspects of operant performance have been published (Francis and Kanold, 2017) and various testing paradigms developed however, behaviors that can be observed with autoshaping rather than hand-shaping clearly demonstrate how the animal can developed unwanted associations. A detailed presentation of reinforcement schedules is well outside the scope of this manuscript and readers are referred to various psychology textbooks for further information (Saini et al., 2016).

## 6 Avoidance Procedures

Avoidance procedures are methods by which to evaluate an organism’s decision to approach or avoid a certain situation. Avoidance of genuinely threatening stimuli or situations is a key characteristic of adaptive fear however, if avoidance loses its adaptive value it may evolve into maladaptive responses which are characteristic of multiple mental disorders (Krypotos et al., 2015). They can be employed to demonstrate a preference or to assess learning and memory. This can apply to multiple tests that involve some aspect of “anxiety-like” behavior (La-Vu et al., 2020) and evaluated with two distinct learning frameworks, passive or active avoidance (Branchi and Ricceri, 2013).

### 6.1 Elevated Mazes and Light/Dark Preference

Assessments in elevated mazes and light/dark boxes are conducted under bright light conditions and based on the rodent’s proclivity toward dark enclosed spaces and unconditioned fear of open spaces. Elevated mazes in configurations that allow for closed arms and open arms are often used to assess the relationship between exploratory behavior in a novel environment and fear induced by a novel stimulus (Montgomery, 1955; Wall and Messier, 2001; Walf and Frye, 2007; Schneider et al., 2011; Casarrubea et al., 2014). This holds for performance in a light/dark box in their configuration to allow the animal to explore the new environment and select a preference and thus, the behavior is based on proclivity toward dark and enclosed spaces and an avoidance of the open areas. The assessment of anxiety behavior of rodents is made by the ratio of time spent on the open area relative to the closed area within a trial.

### 6.2 Passive Avoidance

Passive avoidance is a fear-motivated test used to evaluate associative learning and memory. It is a type of conditioning in which the animal/individual must withhold an explicit act or response that will produce an aversive stimulus. An animal is required to learn to withhold a normally preferred response such as moving from a brightly illuminated large chamber into a smaller dark chamber or moving from an elevated platform to a lower floor. A standard paradigm involves the animal being placed in the non-preferred side of the apparatus and a timed session begins. The animal is allowed to explore the apparatus freely and, upon the first entry in the normally preferred dark side, a noxious event (e.g., scrambled foot shock, blast of air, loud noise, temperature extremes) is initiated from which the animal cannot escape. The animal is allowed to remain in the dark side for up to 15 s to allow for consolidation of the event yet not long enough to diminish reinforcer value. The animal undergoes two or three one-trial sessions with a minimum 24 h inter-trial intervals. To prevent extinction of the behavior, the adverse stimulus remains as a reinforcer during these trials. The strength of the learned association is indicated by an increase in latency to leave the “safe” chamber and enter the “non-safe” chamber, time spent in each chamber, and number of crossings. The strength of the association can be examined by eliminating the adverse stimuli and measuring the number of trials required for the animal to return to a pre-training latency to enter the “non-safe” side, e.g., extinction.

### 6.3 Active Avoidance

Active avoidance is a fear-motivated test used to evaluate associative learning and memory (Diehl et al., 2019). Active avoidance is considered a type of operant conditioning in which a specific act prevents or postpones the delivery of an aversive stimulus. Within the experimental arena, this normally follows a discrete-trial procedure as animals learn to associate a conditioned stimulus (light, tone) with the delivery of an unconditioned negative stimulus (e.g., shock) and to move to a safe location to avoid (avoidance response) or escape (escape response) the negative stimulus. A standard paradigm involves the animal’s being placed in the test apparatus comprised of two chambers and allowed to freely explore. A training trial is initiated by the delivery of a cue (light) followed within a defined time interval by the presentation of a second cue (tone) paired with a negative stimulus. The animal can escape the negative stimulus by moving from the “non-safe” environment upon the delivery of the second cue and shock (escape) and as the association between the cue (tone, conditioned stimulus) and the shock (unconditioned stimulus) develops, the shift in locations occurs with the first cue (avoidance). Acquisition is demonstrated with an increase in avoidance responses and decrease in escape responses over the session. Avoidance latency and number of escape losses are recorded. The number of escape losses (failing to make a response during the full shock delivery period) can reflect an absence of learned association between cue and shock or an increase in fear-related freezing behavior. Active avoidance can be evaluated using various test paradigms, 1) one-way, with actively moving from one location to another; 2) two-way, with actively moving back and forth between two locations with appropriate cues; 3) three-way, with a choice between two locations to escape or avoid. As with any task requiring motor performance and latency, deficits in motor ability or alterations in stress response can influence the outcome in that with the inability to move rapidly the animal will experience multiple no-escape trials or display a frozen fear response. Thus, if an animal is showing escape losses over multiple trials of a session observation of the nature of that response is required.

## 7 Exploratory Behavior/Novelty Tests: Novel Object Recognition, Social Recognition, Social Novelty

There are a variety of tests based on an animal’s natural propensity toward novelty that evaluate an animal’s ability to recognize a previously presented stimulus or to adapt (habituate) to a novel environment. Additionally, they can be used to examine the social motivation drive in rodents (Netser et al., 2020). For novelty, stimuli can be an inanimate object, another animal (social recognition), or changes in the environment. When animals are exposed to a familiar object and a novel object, they frequently approach, and spend more time exploring the novel object than the familiar object, suggesting recognition memory of the familiar object (Antunes and Biala, 2012). The proportion of time spent and number of contacts with each object are recorded over a defined period. This concept can be applied to social recognition of another animal (Mathiasen and DiCamillo, 2010). As with any endpoint that relies on activity, deficits in motor function or activity level and exploration can influence the outcome in this paradigm. Although novel object and social recognition paradigms have been used with greater frequency in assessing the interactive nature of an animal with its environment, application of these behavioral paradigms to assessing chemical-induced neurotoxicity is limited (see Gulinello et al., 2019).

## 8 Experimental Design and Data Analysis

**FIGURE 2:**
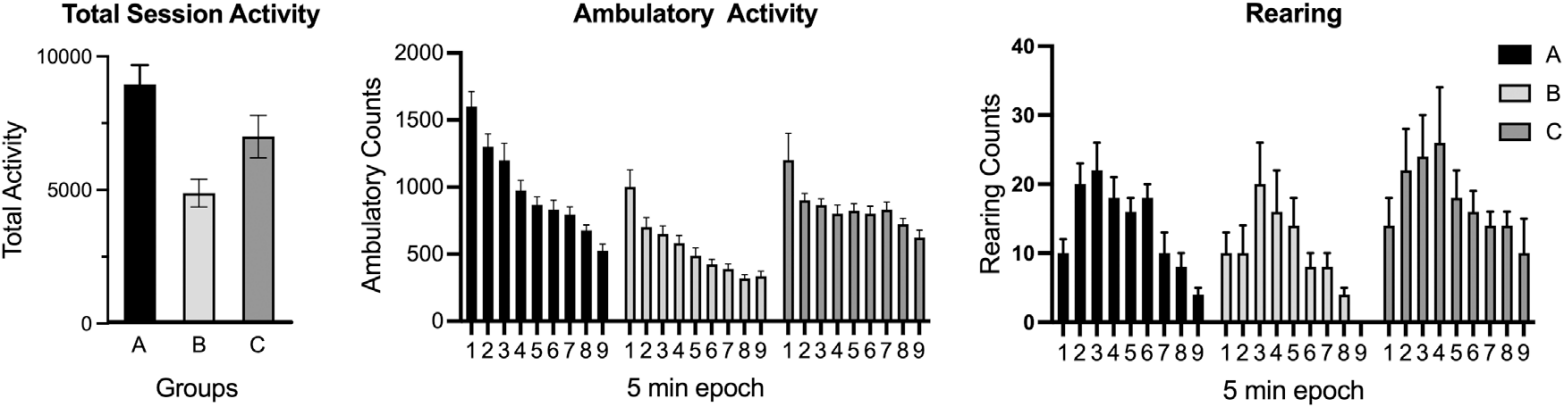
Open field motor activity. Representative data set of activity across an entire session (45 min) and the distribution pattern over 5 min epochs for ambulatory activity and rearing in 75 days old male (n = 20). Data was collected using a commercial device (42 × 42 cm) with photocell detectors (0.32 cm diameter) spaced 5 cm from floor and 1.27 cm linearly apart around the chamber. Horizontal activity photocells were set at 1 inch from arena floor and rearing photocells empirically determined and set at 4 inches. Individual animal time series were examined for extremely high or low performing animals. To meet the normality and homogeneity of variance assumptions of parametric tests. Activity measures over epochs were analyzed using a repeated measured analysis of variance (RM ANOVA) main effects of time (epochs), group, and the interaction between factors with an autocorrelated [AR(1)] error structure reflecting correlation between consecutive epochs. The Total Session Activity data suggests that Groups B and C were less active than Group A. Examination of the components driving Total Activity (Ambulation and Rearing) one gains a better view of what is driving the total session activity differences. In both Group A and B, while Group B is lower the overall pattern is similar and show acclimation. In Group C however, there is a suggestion that the animal initially respond differently to the novel environment and that exploratory activity may be delayed and within the time frame acclimation was not evident but suggested.

**FIGURE 3:**
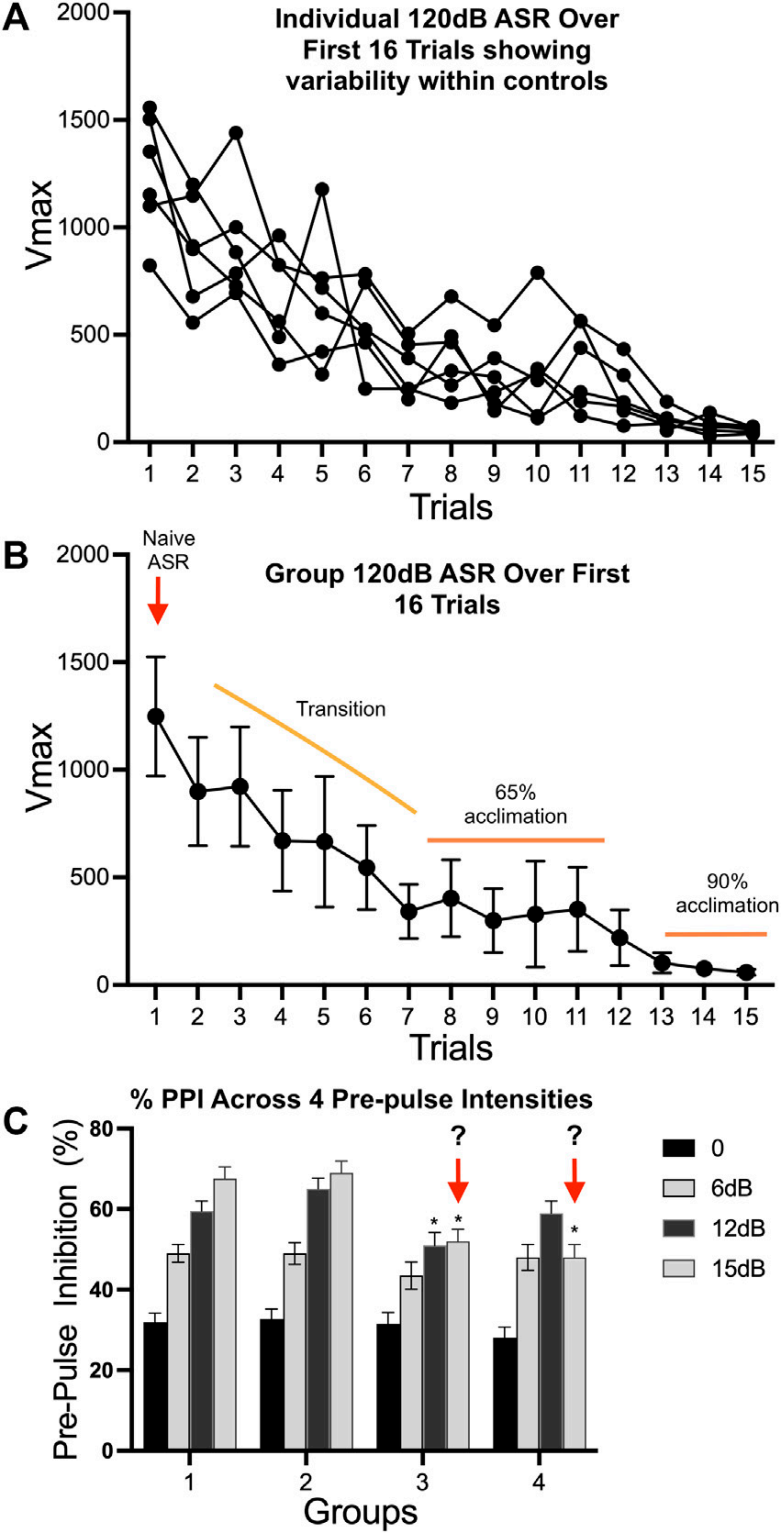
Auditory startle response and prepulse startle inhibition. Representative data for **(A,B)** auditory startle response (ASR) and **(C)** prepulse startle inhibition (PPI) of 90-days old male rats. The ASR is used to assess the integrity of a sensory-evoked motor reflex response and the habituation of response to the startle stimulus. **(A)** represent individual rat ASR (Vmax) to 120 dB across the first 16 trials showing data for individual responses and **(B)** represents this data as a group response. Note that the first ASR trial resents the naive response and often considered the accurate measure of a startle response. The first 10 trials represent a transition of the response, followed by a lower response level plateau and a subsequent full acclimation to the stimuli. The measured startle response is typically log-normally distributed across pulse types (Csomor et al., 2008) and often the median response over a block is more robust measure than the mean. Habituation as demonstrated in **(B)** was evaluated by fitting a RM ANOVA model to the median 120 dB responses across the session with trial (or trial block), treatment, and trial (block) by treatment interaction as factors and accounting for temporal correlation using an autocorrelated [AR(1)] error structure. Alternatively, habituation can be calculated as a ratio of the median of the last block of 120 dB trials to the 1st initial ASR trial or average of first 3 trials. **(C)** Representative patterns of PPI. Reflex modification of the ASR is examined by the delivery of a subthreshold (not producing an ASR) stimulus at a defined auditory level above background (65 dB) prior to delivery of the supra-threshold startle stimulus. The session included one initial 120 dB trial followed by a series of blocks of trial types. Block 1 was comprised of 5,120 dB trials; Blocks 2 and 3 were comprised of 31 trials [2 no-stimulus trials, 6 acoustic startle stimuli (40-msec null period followed by 40-msec 120 dB pulse) trials alone, 18 prepulse stimulus trials (40-msec null period followed by 20-msec prepulse of 3, 6, 12, and 15 dB above a 65 dB background), followed by a 100-msec null period and a 40-msec 120 dB pulse; for an entire recording period of 200 msec] presented in a random order, followed by two additional blocks of 5,120 dB trials. Individual differences require that PPI be calculated relative to the individual 120 dB response prior to averaging across groups. It can be calculated for each pre-pulse intensity as the percentage of the median 120 dB response obtained across Blocks 2 and 3. Negative %PPI values are set to 0%. PPI was analyzed using ANOVA with dose and prepulse type as factors. Group 1 are naïve adult rats. Group 2 represents a pattern of hyper-responsiveness. Group 3 represents a pattern of diminished gating and inhibition at the highest prepulse stimuli. Group 4 represents a pattern of lower inhibition at the 12 dB level suggestive of diminished reflex. (?) indicates the question of whether this represents inhibition or that 77 dB stimuli was not a subthreshold intensity.

**FIGURE 4:**
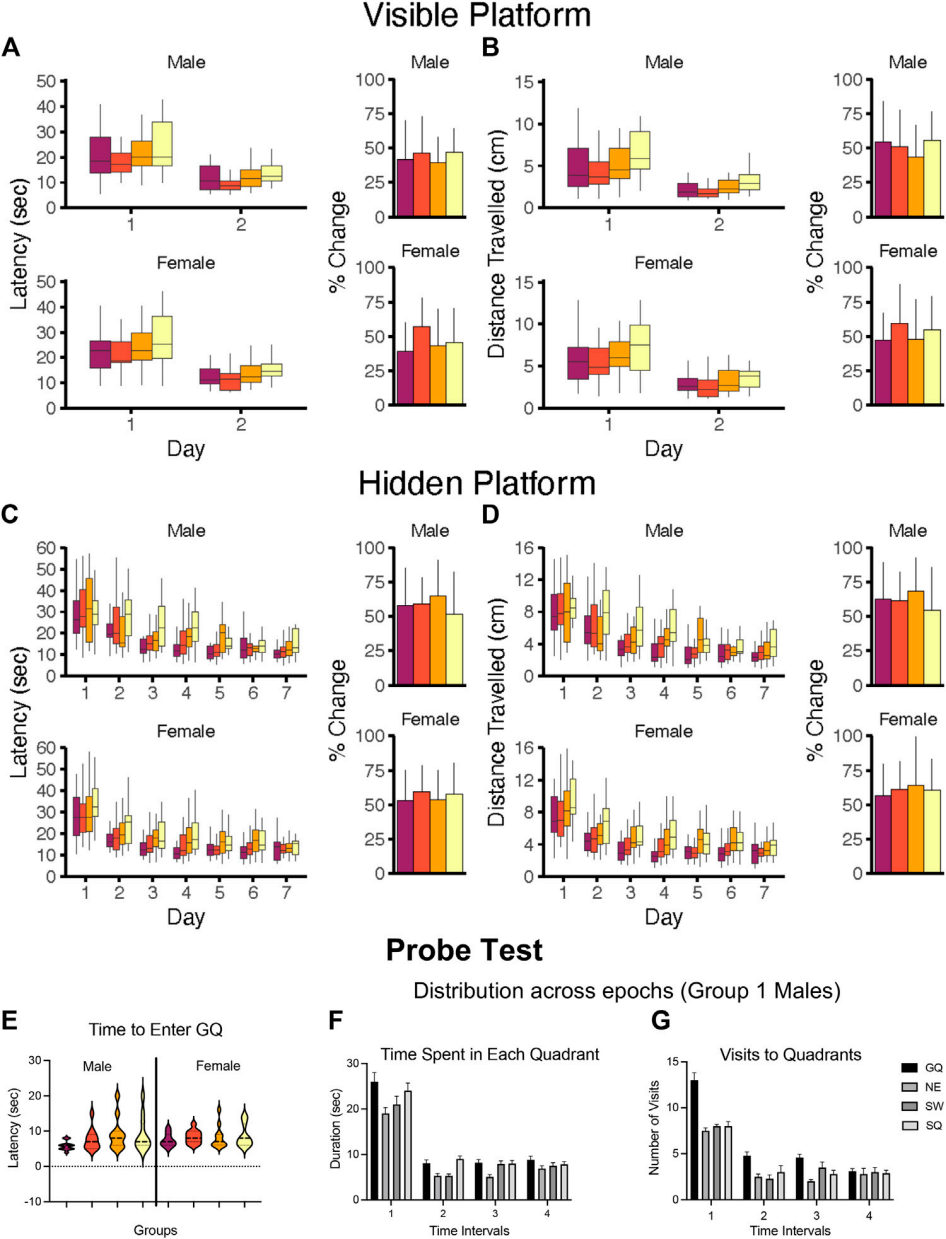
Morris Water Maze. Representative data set of MWM Visual Platform (Cued) and Hidden Platform (Spatial) performance in young adult (75 days of age) male or female Sprague Dawley using a video-imaging (Ethovision XT). A circular black plastic tank (183 × 62 cm) filled with water (25°C) to 10 cm distance from the water surface to the lip of tank. **(A,B)** Cued learning (Visible Platform) confirmed the ability of the animals to perform the task. Three trials, 10 min ITI, were run daily for 2 days. Altering start location. Median daily latencies and distance traveled were analyzed for dose effects using Kruskal–Wallis tests due to non-normality of measurements. The % change across the 2 days was analyzed by a Kruskal–Wallis test. **(C,D)** For spatial learning (Hidden Platform), three daily trials, alternating start location, were administered (10-min ITI) for 7 consecutive days reaching criteria (>85% of control animals showing a >30% decrease from initial latency). Daily median latency and distance measures were calculated due to the skewed nature of the distribution. Data medians, quartiles, and extremes of data distributions. Acquisition was determined by RM ANOVA with day and group as main effects. The % change in latency from the first to the last session was analyzed by a Kruskal–Wallis test with Dunn’s multiple comparison procedure and showed a uniform pattern of habituation across groups. **(E–G)** Representative data for Probe Test. **(E)** Violin plots of the initial latency to enter goal quadrant (GQ) showing no significant difference but an increase in variability across groups. **(F,G)** Representative images for Group 1 male rats of **(F)** time spent in each quadrant and **(G)** number of entries into each quadrant. The representation of intervals (1) 0–90 s, (2) 0–30 s, (3) 30–60 s (4) 60–90 s demonstrate the shifting behavior of the animal and the preference for the GQ.

As in any experiment, the experimental design is dependent upon the research question and needs to be established at the beginning of the study and adhered to in each aspect of the study. Strong considerations of the experimental design, conduct of the study, and expectations for comparisons of the statistical analysis is required prior to study initiation. Depending on the comparisons and the logistics of conducting a study, different experimental designs may be employed to allow for counterbalancing all critical variables. A robust experimental design balances known variability in measured variables, sample size, the magnitude of the effect deemed biologically relevant, and the desired level of statistical significance. Once the experiment is complete and data available for analysis, important initial steps include examination of the distributions of measurements, identification of outliers, and inspection for anomalous outcomes (i.e., non-performing animals, missing observations, implausible values). Measures of central tendency should fit the features of the data. For example, for skewed or censored observations, calculations of the mean may be unduly influenced by extreme observations and bias results. If the experimental protocol calls for sessions comprised of trials with a time cut-off (latency to escape), medians of trials may be more appropriate as a daily summary measurement since measurements could be censored at the maximum allowable trial time as the animal acclimates to the task. Summary measures over time periods where habituation is expected, such as trials within a block in the startle response protocol, may also be better described using medians, which are less affected by extreme observations. The data should meet the assumptions of any statistical procedure used. For example, log transforms of daily latency in the MWM can allow use of parametric methods (i.e., ANOVA, RMANOVA). Other assumptions include normality of observations and homogeneity of variance. Reporting of the data should include details of the testing paradigm and all relevant data, not only those found statistically significant. With the available computer software, multiple redundant endpoints may be collected, some which are relevant to the study and some that provide an internal quality control of the paradigm and data. Representations of the distribution of data can be accomplished by providing all individual data points, using violin plots, or representing variance as a 95% confidence interval. Such representation aids in distinguishing between statistical significance and biological relevance. Examples of data to collect and presentation are provided for motor activity (Table 1; Figure 2), startle and PPI (Table 2; Figure 3), and Morris Water Maze (Table 3; Figure 4).

**TABLE 1:** Arena motor activity.

Data Collection	Data Analysis	Data Reporting
5-minute epochs	5-minute epochs	5-minute epoch
Full Arena	RMANOVA
Fine activity (confirm type of activity)	Factors: experimental condition and time	Ambulatory activity
Ambulatory activity (vertical)	Ambulatory activity	Distance travelled
Distance travelled	Distance travelled	Total activity
Ambulatory time	Total activity	
Rearing (horizontal)		
Total activity (vertical+horizontal)	Total session	Total session
	ANOVA	Full Arena
	Factors: experimental condition	Rearing
Total Session	Full Arena	Perimeter
Perimeter	Ambulatory activity	Distance travelled
Distance travelled	Distance travelled	Time in zone
Time in zone	Rearing	Rearing
	Total activity	
	Perimeter	
	Distance travelled	
	Time in zone	

**TABLE 2:** Startle Response/PPI.

Data Collection	Data Analysis	Data Reporting
Per Trial	Startle Response	Startle Response
Peak response amplitude (V_max_)	V_max_ 120 dB first trial	Response magnitude (V_max_) of 1st 120dB trial
Time to maximum response (T_max_)	[ANOVA or Kruskal-Wallis]	Individual 120 dB (V_max_) trials over session
	V_max_ 120 dB across session	PPI
If Available	[RMANOVA]	% Inhibition for each prepulse intensity
Latency to onset of response	PPI	
Rise time of response	% Inhibition (PPI) for each pre-pulse intensity	
	[(120 dB V_max_ − prepulse V_max_)/120 dB V_max_ × 100.] (for each animal)	
	Set negative PPI values to 0.	
	PPI for each prepulse intensity (ANOVA)	

**TABLE 3:** Morris water maze.

Data Collection	Data Analysis	Data Reporting
Visible Platform	Visible Platform	Visible Platform
Time to reach platform (latency)	RMANOVA (averaged daily trials)	Acquisition (across sessions)
Total distance to platform (path length)	Factors: experimental condition and session (day)	Latency
Swim speed	Time to reach platform (latency)	Distance
	Total distance to platform (path length)	Thigmotaxis
	swim speed	Average swim speed
Hidden Platform	Hidden Platform	Hidden Platform
Time to reach platform (latency)	RMANOVA (averaged daily trials)	Across sessions
Total distance to platform (path length)	Factors: experimental condition and session (day)	Time to reach platform
Time spent floating (% trial duration)	Time to reach platform (latency)	Total distance to platform
Percent thigmotaxis (perimeter) time	Total distance to platform (path length)	% Time spent floating
Thigmotaxic tendency (proportional distance traveled)	Time spent floating (% trial duration)	Percent thigmotaxis time
	Percent thigmotaxis (perimeter) time	Thigmotaxic tendency
	Thigmotaxic tendency (proportional distance traveled)	
Probe Trial	Probe Trial	Probe Trial
1st entry goal quadrant	ANOVA	1st entry goal quadrant/platform zone
Latency	1st entry goal quadrant	Latency
Distance travelled	Latency	
1st entry platform zone	Distance travelled	Distance
Latency	1st entry platform zone	# Platform crossings
Distance travelled	Latency	# Platform zone entries
#Platform-zone entries/crossings	Distance travelled	Swim pattern
Swim pattern	#Platform-zone entries/crossings	Quadrants (total and epochs)
Each quadrant (total and 30-second epochs)	Each quadrant (total and 30-second epochs)	# Entries
Entries	% total (preference for goal quadrant)	Time
Time	Entries	Distance travelled
Distance travelled	Time	
	Distance travelled	
Reversal Learning	Reversal Learning	Reversal Learning
Time to reach platform (latency)	RMANOVA (averaged daily trials)	Across sessions
Total distance to platform (path length)	Factors: experimental condition and session (day)	Time to reach platform (latency)
Time spent floating (% trial duration)	Time to reach platform (latency)	Total distance to platform (path length)
	Total distance to platform (path length)	

## 9 Reverse Translation

Animal models are essential for preclinical research requiring a level of validity of the models for translation to human disorders. Grillon and Ernst put forth the use of re-translational approaches, i.e., conducting model tests in humans for comparison with results of animal studies (Grillon and Ernst, 2016). This includes an effort to maximize the similarity of the measures of response across species. Efforts to model anxiety assessments was reviewed as a possible intermediate bridge between basic and clinical sciences (Grillon et al., 2019). Walz et al. reported that all participants showed some thigmotaxis in an open-field environment (Walz et al., 2016). Agoraphobic patients and patients with high anxiety sensitivity showed a preference along the outer region and less entry into the center of the open field, similar to what is observed in rodent models. The use of virtual environments has allowed for additional assessments of humans on similar learning and memory tasks used in rodents. Using a virtual open field task, Gromer et al. (2021) investigated human movement behavior as analogous to animal studies and showed a strong similarity across species (Gromer et al., 2021). Biedermann et al. (2017) re-translated the elevated plus maze to a human virtual reality test and reported the predicted open-arm avoidance (Biedermann et al., 2017). Using virtual tasks and image analysis species-conserved cognitive mechanism of hippocampal-dorsolateral prefrontal coupling during spatial working memory have been reported (Bähner et al., 2015). Impaired fear extinction has been linked with passive avoidance learning in humans (Cornwell et al., 2013) as well as rodents (Mineka, 1979).

## 10 *In Vitro* Model Systems in Neurotoxicity Assessment

*In vitro* systems are well suited to the study of biological processes in a more isolated context and have been most successfully used to elucidate mechanisms of toxicity, identify target cells of toxicity, and delineate the development and intricate cellular changes induced by toxicants as such, they serve as complementary partners with *in vivo* models in assessing neurotoxicity (Veronesi, 1992; Roush, 1996; Costa, 1998; Harry et al., 1998; Harry and Tiffany-Castiglioni, 2005). The contribution of *in vitro* assays was clearly acknowledged in the U.S. EPA proposed guidelines for neurotoxicity risk assessment (US EPA, 1998b) where it was stated that “Demonstrated neurotoxicity *in vitro* in the absence of *in vivo* data is suggestive but inadequate evidence of a neurotoxic effect. On the other hand, *in vivo* data supported by *in vitro* data enhance the reliability of the *in vivo* result.” With technological advances and the ability to generate a limited number of neural cells from human cell lines or from patient populations, the ability to target examination of mechanisms of neurotoxicity has significantly advanced.

While these systems offer an important approach to address issues of neurotoxicity, they are not devoid of limitations that require attention if these systems are to be put forth as a stand-alone approach to evaluate potential for human neurotoxicity. The applicability of these systems to address questions related to neurotoxicity and developmental neurotoxicity screening has been reviewed in recent articles (Rosca et al., 2020; Sachana et al., 2021) as have limitations (US EPA, 1994a). Given the focus of the current special issue it is anticipated that many of these model systems proposed for neurotoxicity assessment will be discussed in methodological detail in other articles. Additionally, critical reviews covering the benefits and limitations of such systems in modeling the brain environment and translating to use for human disorders are available (Barbosa et al., 2015; Young-Pearse and Morrow, 2016; Fink and Levine, 2018; Chen et al., 2019; Oliveira et al., 2019; Gordon and Geschwind, 2020; Lee et al., 2020; Slanzi et al., 2020; Eremeev et al., 2021; Franklin et al., 2021; Xu and Wen, 2021). Of equal importance of evaluating their benefits and limitations is the opportunity to mesh the *in vitro* and non-mammalian approaches with the mammalian models to develop a more integrated approach for assessment.

The value of understanding fundamental mechanisms of neurotoxicity has been the basis for developing developmental or disease specific micro-physiological systems. These models include 1) brain organoids which can exhibit a variegate cell composition. 2) micropatterning to address the issue that cells grown in a dish adopt a multitude of shapes building colonies of variable forms and densities (Blin, 2021), 3) the use of biomaterials, any material that has been designed to purposefully interact with individual cells or cell constructs, to model the *in vivo* environment (Roth et al., 2021), and 4) microfluidic systems to engineer the architecture to shape the physical and chemical microenvironment for specific research questions (Nikolakopoulou et al., 2020; Holloway et al., 2021). Some have proposed complicated strategies with a combination of methods (biology to bioengineering) involving fusion of several organoids into “assembloids” containing multiple brain areas, interconnected neurons, glia, and capillaries (Le Cann et al., 2021; Makrygianni and Chrousos, 2021; Xu and Wen, 2021). While these approaches show promise, applicability to human disease or vulnerability becomes rather difficult due to the nascent nature of the field. Because the field is in a state of flux, papers reviewing complex systems often highlight their potential with a cursory mention of limitations (Qian et al., 2019; Le Cann et al., 2021; Xu and Wen, 2021). With the increasing body of data and the number of laboratories incorporating such models and new technologies into their research, the field will have a better knowledge of the models, their benefits and limitations that may affect their translation to clinical problems (Qian et al., 2019; Velasco et al., 2019; Chiaradia and Lancaster, 2020; Marx, 2020; Sarieva and Mayer, 2021). As an example, organoids fail to recapitulate the characteristic human cortical development of progenitor maturation trajectories and the emergence of diverse cell subtypes and areal specification of newborn neurons as well as spatially segregation of molecular signatures (Chen et al., 2019; Bhaduri et al., 2020). In this study, organoids ectopically activated cellular stress pathways that impair cell-type specification. These deficits were alleviated upon the cell transplant into the *in vivo* brain region-specific environment, demonstrating the uniqueness of the *in vivo* environment (Bhaduri et al., 2020).

For assessment of chemical-related neurotoxicity and the potential to induce an adverse effect *in vivo* the benefit/limitation of such systems is dependent upon the research question asked. While the *in vitro* model systems offer great potential, they also raise caution for extrapolation to the whole organism. Many of these systems are proposed for use in modeling features of the mature or developing nervous system such as key developmental processes or cell function and key characteristic associated with neurotoxicity or neurodegenerative disease. How the proposed endpoints reflect, predict, or could be extrapolated to what might occur with *in vivo* exposure is a major concern. This is reflected in the efforts to develop a battery of assays for assessing neurotoxicity using multiple *in vitro* models and incorporating multiple endpoint assessments (Schultz et al., 2015; Heusinkveld and Westerink, 2017; Harrill et al., 2018; Masjosthusmann et al., 2020). How these model systems can be utilized in the field of neurotoxicology will depend on the question at hand and the target endpoint for assessment.

### 10.1 Experimental Design and Interpretation

The experimental design questions for analysis of data from *in vitro* or non-mammalian studies are somewhat like those for *in vivo* studies. To move this approach forward in a meaningful manner there are a few critical steps required. An initial step is to optimize and standardize protocols and details of experimental design to provide guidance of how to evaluate quality of the data set, confirm the unit for analysis (individual well, average of duplicates), and the number of study replications required for confidence in the data. This includes information on the level and directionality of a change considered to be of biological significant. Thus, as screening efforts progress and involve numerous different laboratories, including non-academic laboratories, conducting similar assessments one wants to minimize the chance that in another decade the new approach will land in an identical place as the *in vivo* apical behavioral endpoints. As with *in vivo* methods, while tempting, the more-simple assays may be the easiest to conduct; however, they may be the more difficult to interpret with regards to translation to whole organism effects. Thus, while advocating for *in vitro* and non-mammalian model systems is relatively easy, taking that next step to confirm the various types of validity of the model, the endpoints, and the reproducibility of the system across multiple labs is a more difficult step. To determine what model system and what endpoints should be obtained is somewhat hindered by the vested interest, intellectual and financial, of the existing laboratories, but it is necessary. There is now over a decade of work developing these model systems and screening various chemical libraries, taking advantage of new imaging technology and cell technology as they become available. What has been learned? What models are showing good level of reproducibility? Qualitative or quantitative? What criteria should be set for any study? Are we being courted by technology shinny objects or staying within a relevant biological question? For the likely candidates, the next critical step is to address the issue of predictive validity. For example, for something like neurite outgrowth which is rationalized to represent a stage of brain development, would a similar pattern be observed if the process was examined within the framework of chemoattractant vs. chemorepellent signals as would be present *in vivo*? What would be predicted to occur *in vivo* and how might one model that *in vivo* either developmentally or with repair following injury? There exists knowledge in developmental neurobiology to design such studies. There just needs to be the interest to conduct the studies and the willingness to accept the findings and modify an assay as needed. Such a targeted effort would provide support for interpretation that any disruption observed would be representative of what would occur or be relevant with exposure *in vivo*.

### 10.2 Properties of Chemicals and Exposure

Physical properties of various compounds may prevent a direct evaluation *in vitro*. This may be related to physicochemical properties of chemicals, such as solubility, volatility, pH, binding to components of the culture medium including protein binding, osmolarity, or the need for active metabolites. The issue of properties is well demonstrated in the examination of nanomaterials. The nanoscale of these chemicals drives several high reactivity properties leading to instantaneous encapsulation by a corona. Made of biomolecules, mostly proteins, the corona composition depends *in vivo* on the site of entry (e.g., surfactant proteins in the lung) and the translocations steps (plasmatic proteins in the blood vessels) before being available for potential transport across the blood brain barrier and into the brain parenchyma. This composition may influence biodistribution, cellular binding and uptake, internalization process, and resulting adverse effects. *In vitro*, the corona composition differs as dictated by the culture media. Thus, while effects can be observed *in vitro*, translation to effects in the whole animal can be difficult. Thus, any effort to extrapolate neurotoxicity potential of nanomaterials from *in vitro* studies would require complementary data from *in vivo* exposure.

The other aspect of exposure that does not appear to be addressed in the various efforts to screen chemical libraries is the relevance of the exposure levels. In the absence of experimentally determining the target tissue levels relative to a systemic exposure then even extrapolating effects, dose related or not, from a direct administration of a chemical to cells in culture is in question. Thus, while the argument can be put forward that *in vitro* or non-mammalian tests are faster and cheaper and that a replication study can be conducted easier than an animal study, if the nervous system never sees the level of a chemical used in culture, or only sees a metabolic product of that chemical, the ability to reproduce an effect *in vitro* has limited value for determining the risks of effects *in vivo*.

### 10.3 Need for a Systems Biology Approach

As representative of chemical exposures to humans, limitations in aspects of inter-organ/tissue communication and effects, interactions with the peripheral nervous system, hormonal (sex, thyroid, stress) influences, and influences of systemic toxicity have been identified. Distinguishing sex specific responses or responses across a diverse population are limited, as is the ability to model chronic exposures. With regards to life stage susceptibility, the critical influence of maternal factors (e.g., inflammatory status, hormones, organ system dysfunction, placenta integrity), unique events in adolescence, or susceptibility in the aged are not represented in many of the proposed models. Similar limitations can be raised for environmental exposures that contribute to neurotoxicity with alterations in cardiac function, kidney function, and actions through the microbiome. These imitations are only presented to foster the appreciation and understanding of what each type of system, *in vitro* or *in vivo*, can offer to our understanding of neurotoxicity. There is no question that the power of *in vitro* models lies in their ability to address mechanisms of toxicity and possible targeted cell-cell interactions and to refine and focus specific questions for examination *in vivo*. The question now is, how does the field bring this back to a systems biology approach for an integration rather than a dicotomy of one versus the other?

#### 10.3.1 Endocrine Disrupting Chemicals

One clear example of the need to approach neurotoxicity in a systems biology manner is the evaluation of chemicals that may have disrupting effects on the endocrine system. The classification of a chemical substance as an endocrine disrupting compound (EDC) requires identification of its adverse effect, endocrine mode of action, and link between both. This requires both the integrated circuitry of the central and peripheral nervous systems and may involve both direct and indirect modes of action. The indirect mode of action involves changes in circulating hormone levels, which then may affect the organization or activation of neural structures involved in the expression of the behavior. The circulating levels of hormones may be modified by 1) dysregulation of the hypothalamic/pituitary axis controlling the target peripheral organ at the origin of hormonal synthesis and liberation, 2) direct impact on hormonal synthesis and liberation by the target organ, or 3) changes in the transport/metabolism of the hormone. Thus, it seems difficult to consider modeling *in vitro* the reciprocal dialogue between the brain and a distant endocrine organ, which often involves complex feedbacks involving even several endocrine organs. The ratio of hormonal receptors in a specific cell at a specific stage of life is dependent upon the circulating levels of hormones. Exposure to an EDC can directly affect the neural structures underlying the behavior by interfering with 1) the neural synthesis of hormones such as neural metabolization of testosterone into estradiol by aromatase or conversion of thyroxine into triiodothyronine by deidoniases, 2) binding to and activation of hormone receptors, or 3) the expression levels of these hormone receptors. While *in vitro* studies can help to characterize the molecular mechanisms in a specific cell type, experimental *in vivo* studies are still mandatory to identify the adverse effect, the underlying endocrine mode of action, and the link between both.

### 10.4 Definition of Neurotoxicity for *In Vitro* Models

The definition of neurotoxicity applied to *in vivo* studies implicated an “adverse outcome” with rather broad definitions of “adverse”. Unless focused on mechanism of action, the question arises as to how will neurotoxicity be defined for *in vitro* assessments? If any change seen is considered “adverse” one may raise a concern for false positives with descriptive endpoints (death, proliferation, morphology, activity) given the non-natural nature of culture systems. Additional questions for the interpretation of *in vitro* “neurotoxicity” include issues of neurotoxicity specificity versus cell toxicity, whether the effect or dose level effect was generalized or specific to the cell type or even for how the cell was obtained (primary, stem cell, inducible pluripotent stem cells, cell line, rodent or human). One critical issue in interpreting data from experimental studies is the aspect of human relevant dose and the target tissue dose. *In vivo*, the biological processes that determine the amount of chemical that will reach the brain are intact. In the absence of accurate target tissue dose information, levels used *in vitro* may not be relevant to *in vivo* exposure and comparison of dose across multiple compounds may also not be relevant. One of the more concerning questions relates to if a “neurotoxic” effect is observed *in vitro* but not observed *in vivo*, which finding will be considered representative of an adverse effect? Given that many of the current *in vitro* test systems rely heavily on structural endpoints, integrating those with molecular and biochemical changes and with comparable *in vivo* changes can contribute to answering these questions. In addition, the identification of a common underlying mechanism associated with the outcome would lend significant support for any conclusion of neurotoxicity.

## 11 Summary

In the field of cell biology and neuroscience the interchange of data obtained from *in vitro*, non-mammalian organisms, and rodent studies has served to facilitate an understanding of the fundamental biology and mechanisms associated with normal functions and alterations in the nervous system. Such approaches have their greatest potential to inform on basic mechanistic processes and to refine specific experimental questions to be addressed in the whole animal. The constant interchange of findings across model systems and use of the “most relevant” system for the question at hand offer the likelihood of translation to humans. A strong base in fundamental research, model validation, and species specificity for the question at hand is required prior to considering an applied screening utility of such models. Expectations placed on any model system needs to be realistic and within the realm of neurobiological limitations. The approaches are complementary, not competitive, and all data serve to provide a weight-of-evidence for adverse outcomes following any specific manipulation or exposure. Thus, there is a need for multiple tools and model systems and the integration of data across multiple toxicity endpoints to evaluate neurotoxicity.

## 12 Scientific Impact

The power of experimental models lies in their complementary nature. An appreciation of the complexity of the nervous system and the need to integrate anatomical, mechanistic, and apical endpoints is required. Integration of multiple model systems and recruitment of a multi-disciplinary approach are needed to support advancement. This comes with partnerships and scientific appreciation of the value of all approaches to reach a common goal. This article highlights how one might re-view behavioral studies and reiterates cautions of relying on non-mechanistic *in vitro* models to predict apical outcomes. The goal is to bring a better understanding of these approaches and how they can be used to meet current and future challenges. Integration is a hard but necessary next step. Not for the distant future but at this timepoint when addressing the various issues and the biases is still possible.
